# Gyroid Labyrinth of Supertwisted Double Helices in a Liquid Crystal Polymer

**DOI:** 10.1002/anie.202522314

**Published:** 2025-12-04

**Authors:** Yumin Tang, Yi‐nan Xue, Shu‐Gui Yang, Ruibin Zhang, Feng Liu, Xiangbing Zeng, Goran Ungar

**Affiliations:** ^1^ School of Chemical Materials and Biological Engineering University of Sheffield Sheffield S1 3JD UK; ^2^ Shaanxi International Research Center for Soft Matter, State Key Laboratory for Mechanical Behavior of Materials Xi'an Jiaotong University Xi'an 710049 China

**Keywords:** AFM, Chirality, Depolarized fluorescence, SAXS/WAXS, Self‐assembly

## Abstract

A liquid crystal (LC) polymethylsiloxane (PMS) with rod‐like aromatic side‐groups attached via an alkylene spacer and bearing three n‐dodecyl end‐tails is found to form an unusual cubic structure. In a normal LC double gyroid (DG), the two chiral subspaces, one each side of the G‐surface, are occupied by one network each. Here each such network is split into two aromatic strands that wind around the central polysiloxane bundle, forming a double helix, resulting in a four‐network gyroid (4NG). While in previous normal LC DGs the network twist was assumed to follow that of the subspace, in 4NG the twist sense of the double‐helix is opposite to that of the subspace., i.e., while a right‐handed subspace twists by +70.5° between junctions, the double‐helix “supertwists” by −109.5°, and the opposite is true for the left‐handed subspace. Detailed analysis by X‐ray diffraction, DSC, and depolarized fluorescence (DF) shows a gradual but significant reversible change in the degree of mixing between the aromatic side groups and the polysiloxane backbones at 120 °C–130 °C in 4NG. Also, a significant increase in the system mobility starts only at ∼40 °C above the melting point, indicating persistence of local double‐helical segments even in the melt.

## Graphical abstract

A side chain liquid crystal (LC) polymethylsiloxane (PMS) forms an unusual four‐network gyroid (4NG) structure. In each of the two chiral subspaces, separated by the gyroid minimum surface, there are two networks of aromatic moieties instead of one. These wind around the central polysiloxane bundle, forming a double helix. Furthermore, the double helices are found to supertwist, their chiralities being opposite of those of the subspaces they occupy.

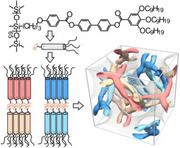

## Introduction

Among the many self‐assembled supramolecular structures in soft matter, the double gyroid (DG) is ubiquitous,^[^ ^1^ ^]^ found in lyotropic liquid crystals (LCs),^[^ ^2^ ^]^ block copolymers,^[^ ^3^, ^4^, ^5^ ^]^ and thermotropic LCs.^[^ ^6^, ^7^ ^]^ There are three main classes of thermotropic molecules that form the gyroid: rod‐like with flexible end‐chains (or polycatenars),^[^ ^7^, ^8^, ^9^, ^10^, ^11^, ^12^ ^]^ rod‐like with side‐chains,^[^ ^13^, ^14^ ^]^ and taper‐shaped dendrons with multiple end‐chains.^[^ ^15^, ^16^ ^]^ DG can be used in photonics,^[^ ^17^, ^18^ ^]^ as 3D electronic or ionic conductors,^[^ ^16^, ^19^ ^]^ in membranes^[^ ^20^ ^]^ and as templates for well‐defined porous ceramics for use in separation or catalysis.^[^ ^21^ ^]^ As far as we know, there has been only one report of the gyroid phase in a polymer outside the block and star copolymer field.^[^ ^22^ ^]^

The DG structure is 3D periodic with cubic symmetry and space group $Ia\bar{3}d$. In a typical representation, its structure contains two infinite interpenetrating networks separated by an infinite 3D periodic surface of minimum curvature (the G minimum surface^[^ ^23^ ^]^). Three network segments join at planar junctions, each of which has three‐fold rotational symmetry. The structure is bicontinuous, meaning that both the networks and the minimum surface are continuous in space, rather than discrete.

Even though DG has mirror symmetry, hence is achiral, its two subspaces as divided by the G‐surface are chiral, with symmetry *I*4_1_32. They are mirror images of each other. We term this antiferrochirality, as the system is only achiral because the chiralities of the two subspaces cancel.^[^ ^24^ ^]^ It is also worth noting that for photonic and plasmonic applications a chiral single network structure (“single gyroid”) is preferred,^[^ ^17^, ^18^, ^25^, ^26^ ^]^ as is often adopted by biological systems such as butterfly wings.^[^ ^27^ ^]^

In the DG phase formed by polycatenar compounds, the molecules assemble into columnar segments of the networks with their rod‐like cores normal to the column axis. The network segments are surrounded by flexible end‐tails, which fill the space between the networks and around the minimum surface.^[^ ^6^, ^28^ ^]^ While the intrinsic chirality of each network should be expected to play a role in the way the rigid cores of the molecules pack, only recently has it been proposed that the orientation of the molecules should twist left or right along the network segment. It is thought that long‐range propagation of uniform twist sense is supported by the need for all three branches at each junction to be isochiral in order to avoid molecular clashes at the junction.^[^ ^29^, ^30^ ^]^ It was further assumed that the hand of the molecular twist coincides with the hand of the network itself, which is 70.5° between adjacent junctions in one network and −70.5° in the other. This model is supported by a recent study by resonant scattering.^[^ ^31^ ^]^

Polycatenar molecules form two other bicontinuous phases, both chiral even if the compound is achiral. One is cubic with a bigger unit cell. It is proposed that it contains three interpenetrating networks, all of them isochiral, with space group *I*23.^[^ ^32^ ^]^ There is also an alternative model for this phase.^[^ ^33^ ^]^ The other bicontinuous phase is tetragonal, so‐called “Smectic‐Q”, containing two isochiral networks.^[^ ^34^ ^]^ Chiral compounds prefer to form one of the latter two phases, but recently we found that a strongly chiral compound can form a chiral DG phase, where the twist of molecular orientation in both networks is of the same sense, either left or right, depending on the hand of the molecules. In one network, the molecular twist between junctions is +70.5°, but in the other, it is a “supertwist” of +109.5°, instead of −70.5°.^[^ ^35^ ^]^ In an ABC triblock copolymer where the A block is chiral, it has recently been reported that the chiral block prefers to occupy one of the chiral subspaces over the other, leading to a DG phase with net chirality.^[^ ^36^ ^]^

**Figure 1:**
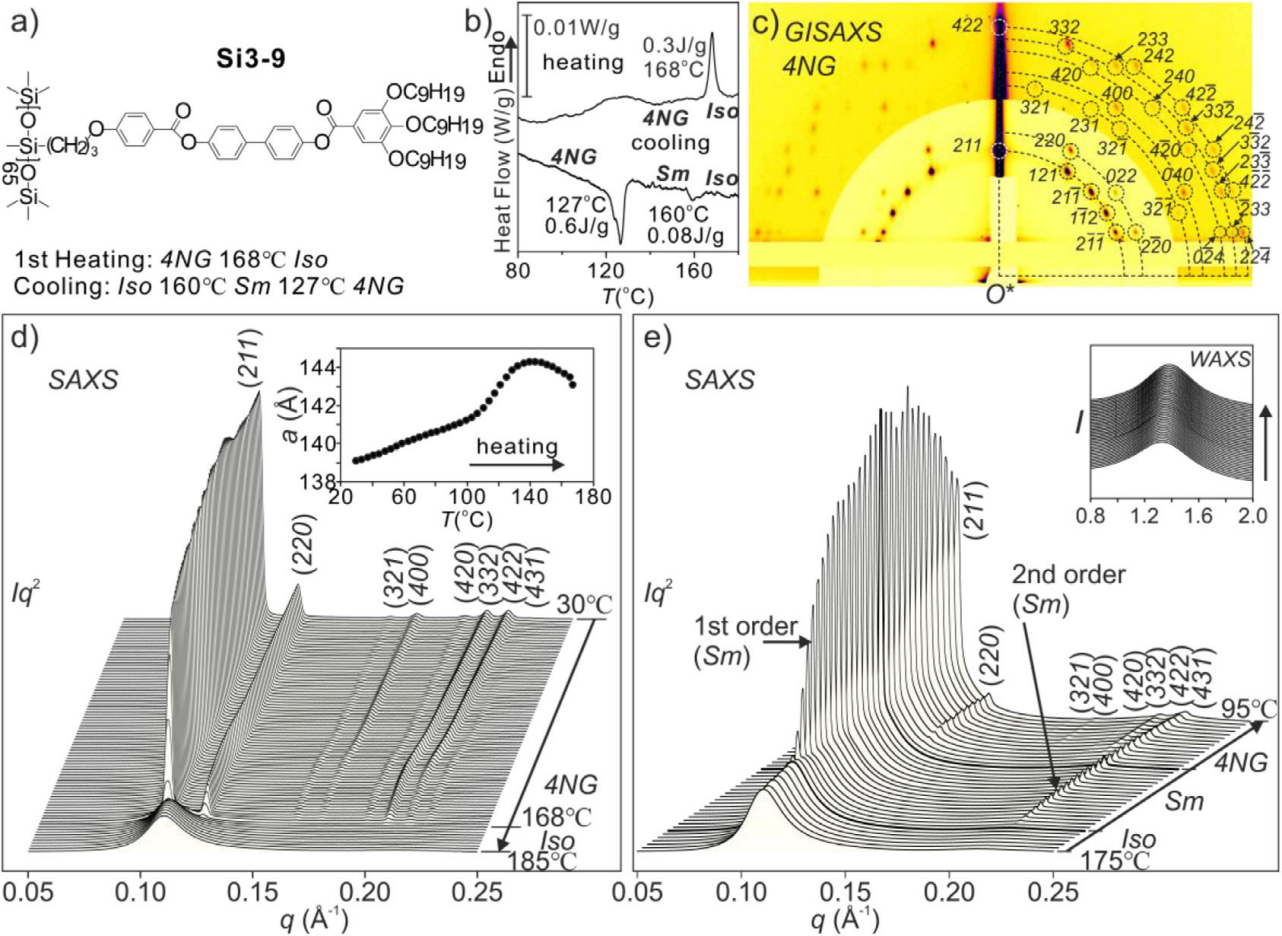
a) Chemical formula of **Si3‐9** and its phase sequences on heating and cooling. b) DSC thermograms of **Si3‐9** on heating and subsequent cooling, both at 5 K min^−1^. c) GISAXS pattern of the cubic phase of a spin‐coated and dried sample recorded at 95 °C in the cubic (4NG) phase, with peaks marked with their Miller indices. d) Powder *SAXS* diffractograms recorded during 5 K min^−1^ heating. Before the DSC heating scan in b) and the SAXS heating scan in d), the sample had been cooled at 0.2 K min^−1^ from 140 °C. Inset in d): the change of lattice parameter *a* of the 4NG phase as a function of temperature. e) SAXS traces recorded during cooling from Iso to cubic (4NG) via Sm; inset: diffuse WAXS peak recorded simultaneously.

In the current study, we examine the formation of the DG phase in an LC polymer, **Si3‐9**, with a polymethylsiloxane (PMS) backbone and side‐groups containing a rod‐like aromatic mesogen connected via a propylene (*m *= 3) spacer and bearing three nonyloxy (*n *= 9) chains at its end (see Figure 1a). Similar polymers bearing pentyloxy and dodecyloxy end chains have previously been shown to display a smectic and a columnar LC phase, respectively.^[^ ^37^ ^]^ A detailed structural study of another polymer of this type, **Si9** **‐** **12**, recently found it to form an antiferrochiral 3D columnar LC with an equal number of left‐ and right‐handed chiral columns. In each column, the aromatic mesogens form a double helix, the two strands winding around each other and around the central bundle of PMS main‐chains.^[^ ^38^ ^]^ Our present study of the gyroid in **Si3‐9**, as laid out in detail below, shows that the columnar segments between network junctions are also double helices and that they all “supertwist” counter to the twist of the subspace that they occupy, bounded by the minimum G‐surface. This results in what we call a “four‐network gyroid” (4NG) structure. The rationale is discussed for this surprising behavior and for the structure of bicontinuous phases in general. Included in this report are also the behavior of the material confined to ultra‐thin film and the finding that serious supramolecular disassembly starts only well above the melting point.

## Results and Discussion

The synthesis of **Si3‐9** is described in Section S2. All characterization procedures, physical, chemical, and structural, are described in Section S1.

### The Four‐Network Gyroid

Powder small‐ and wide‐angle X‐ray scattering (SAXS and WAXS) recorded during a 5 K min^−1^ heating of a very slowly cooled (0.2 K min^−1^) sample is shown in Figure 1d. Only one phase, a bicontinuous cubic with space group $Ia\bar{3}d$, is seen in the entire mesophase temperature (*T*) range, i.e., up to isotropization at *T_i _* =* *168 °C. We refer to this phase as 4NG for reasons that will become obvious below. However, on cooling (Figure 1e), a metastable smectic (Sm) phase forms first from isotropic (Iso) melt, transforming to the cubic around 127 °C. It should be mentioned that a less perfect cubic phase, such as that obtained on faster cooling, on subsequent heating transforms to the Smectic a few degrees below *T_i_*. The brief appearance of the Smectc will be noticeable in Figure 6.

Indexing of the powder SAXS and grazing incidence (GISAXS) peaks of the cubic phase is given in Figure 1c–e and Tables S1–S4. As expected from a cubic, the phase is optically isotropic when examined under a polarizing microscope, unlike the Sm phase from which it emerges on cooling (Figure S5). Miller indices of the diffraction peaks obey the rules *hkl*: *h+k+l *= *2n* and *hhl*: 2*h*+*l *= 4*n*, indicating a body‐centered lattice with space group $Ia\bar{3}d$. Regarding intensities, (211) and (220) are still the dominant peaks, as is generally the case with DG structures observed before. The LC nature of the cubic and *Sm* phases is supported by the simultaneously recorded WAXS, with a broad scattering peak centered at q ∼1.4 Å^−1^, corresponding to d‐spacing ∼4.5 Å if the Bragg equation is used. The WAXS peak does not differ much from that of the Iso melt (inset in Figure 1e).

**Figure 2:**
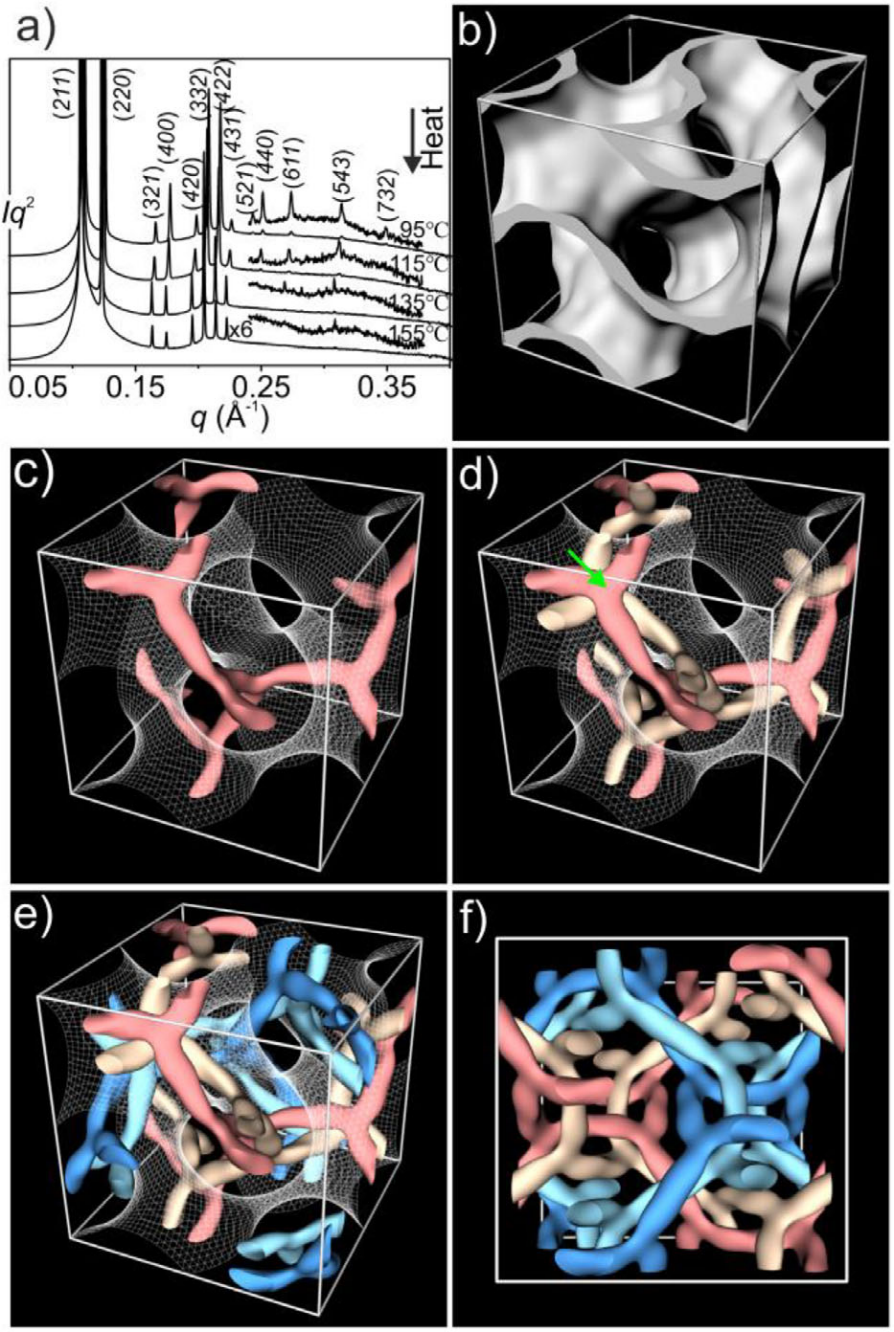
a) SAXS diffractogram of the 4NG phase of **Si3‐9** at four different temperatures from 95 °C to 155 °C. b) Low *ρ* regions (enclosing 18% of the total volume and ∼1/3 of the aliphatic end‐chain volume) of the reconstructed map at 95 °C. c) One of the four networks with high *ρ*. In panels c)–e) the ideal G‐surface is outlined as a net. d) Two interwoven networks of high *ρ* regions, colored in red and gold, respectively, on the same side of the minimum surface. The $\bar{3}$ symmetry at the junction is guided by the green arrow. e) Four high‐*ρ* networks, 11% of the total volume, and 25% of the aromatic parts of **Si3‐9**. f) Top view of the four networks.

At 95 °C, 13 Bragg peaks of the cubic phase, up to (732), can be identified in SAXS (Figure 2a). The lattice parameter *a* = 141.6 Å. The electron density (*ρ*) map has been reconstructed based on diffraction intensities listed in Table S1. The phase combinations have been chosen so as to achieve clearly defined high and low *ρ* regions corresponding, respectively, to the aromatic cores and aliphatic end‐tails of the molecules (Section S7). In the chosen map, this is evident in the presence of two clear peaks in the volume versus *ρ* histogram near both the high and low *ρ* limits (Figure S8). As expected, the low*‐ρ* region, corresponding to the aliphatic tail ends, closely follows the minimum G‐surface (Figure 2b). We will refer to the spaces on each side of the G‐surface as subspaces.

Interestingly, the highest *ρ* 11% of the total volume (or a quarter of the aromatic volume), enclosed by the isoelectron surfaces in Figure 2c–f, forms four instead of two networks found in the normal DG phase. There are two networks (red and gold) in one subspace and another two (blue and light blue) in the other. The two networks, or strands, of a network pair (e.g., red and gold, Figure 2d) are seen winding around each other tightly. The individual networks have a reduced symmetry of *I*2_1_3 only (Figure 2c), while the pair as a whole has symmetry *I*4_1_32 (Figure 2d). It should be noted that even though the subspace occupied by the red and gold networks is left‐handed, the winding of the network segments around each other is right‐handed. The opposite is true for the two blue networks in the other subspace. Henceforth, we shall refer to the new gyroid phase as four‐network gyroid, or 4NG.

**Figure 3:**
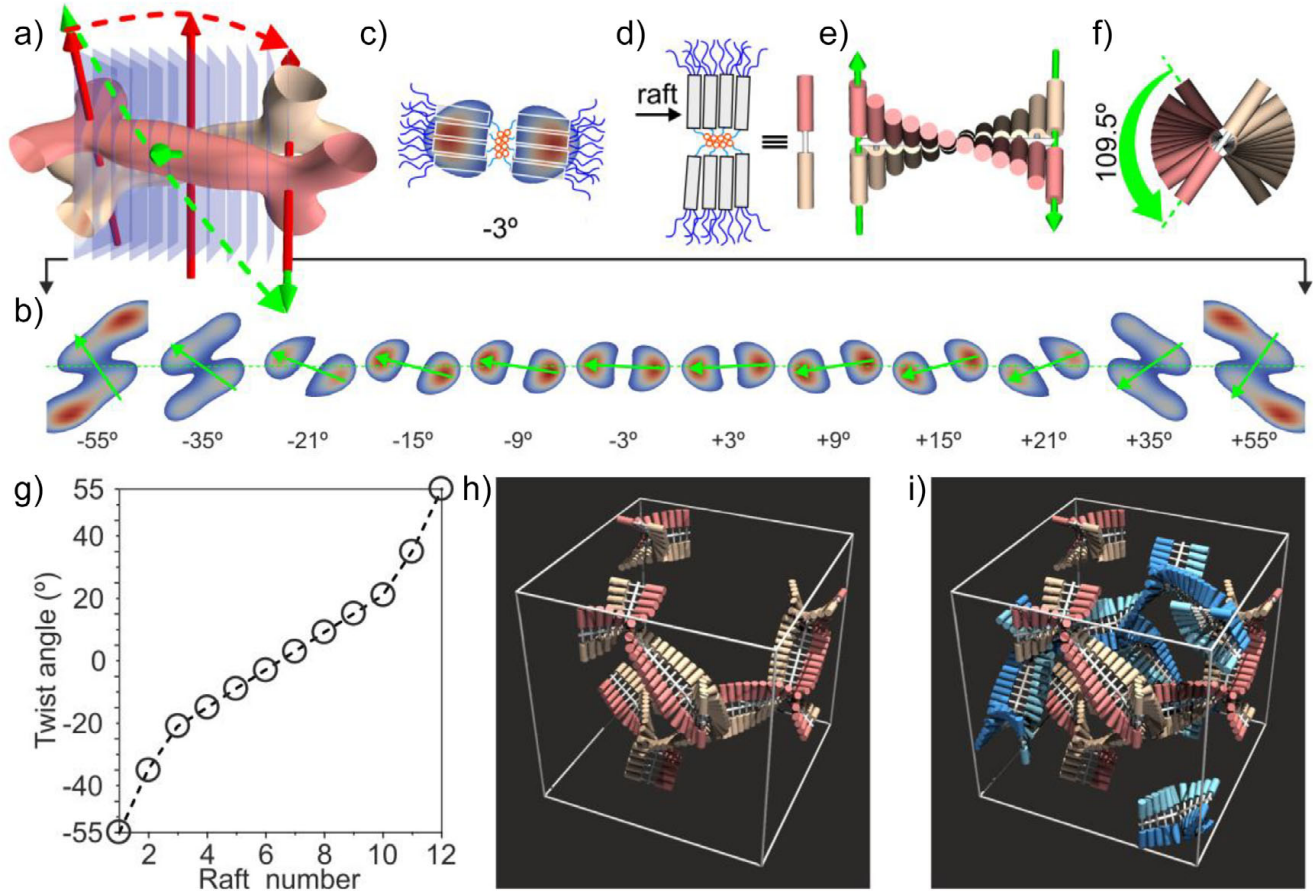
a) Slices (in semi‐transparent blue) of the two strands of a segment of the left‐handed network. The green dashed line, connecting the green arrows, traces the 109.5° twist of the molecules between the junctions. The red dashed line traces the twist of the network itself, defined by the −70.5° dihedral angle between the junctions (red arrows). b) 12 slices from a) showing the high‐*ρ* regions. The small green arrows connecting the two *ρ* maxima in each slice define the azimuthal angle, listed below each slice and plotted versus slice number in g). c) Schematic **Si3‐9** side groups superimposed on the −3° slice in b). d) About four mesogens form a raft at each side of the backbone bundle. A raft pair model is further simplified as a dumbbell with its two rods in colors of the four networks. e) Molecular rafts supertwist to form a double helical segment between two 3‐way junctions; f) top view of the supertwisting column, with a twist angle of 109.5°. h) Dumbbell model of the unit cell, showing two interwoven networks colored red and gold, respectively. i) Dumbbell model with all four networks, the other two colored light blue and blue, respectively.

**Figure 4:**
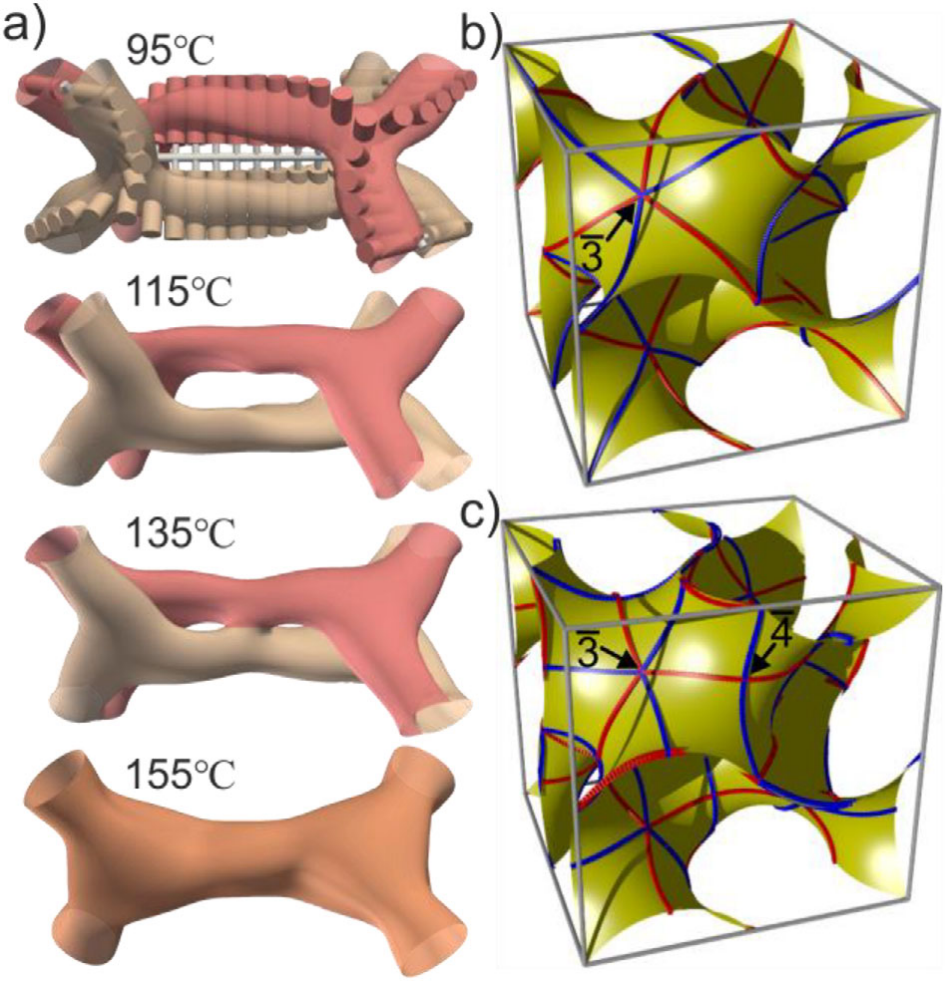
a) Isoelectron surface enclosing the high*‐ρ* regions of a network segment including the two bounding junctions at a series of increasing temperatures. A model of a twisting polymer bundle with two aromatic strands is overlaid on the first *ρ*‐map. With increasing temperature, the two strands come closer and gradually merge as PMS main chains unbundle and mix with the side‐groups. b) and c) The projection of the axes of the side‐group from the network to the minimum surface, mapping the trajectory of the average position of the end tails. Blue lines are from the right‐handed network, and red lines are from the left‐handed. b) Rafts and network twist, the same way, i.e., ±70.5° between junctions. c) Rafts supertwist by ∓109.5°. While the trajectories in b) cross on the minimum surface only at points with symmetry $\bar{3}$, in c) they cross at both $\bar{3}$ and $\bar{4}$ points. In c) the end chains are more evenly distributed on the minimum surface.

A better way to understand the complex tetra‐network structure is by replacing each straight network segment of the normal DG structure with a double‐helical column. In order to examine the double‐helical segments in more detail, we have taken slices of the electron density map along one such segment between two neighboring junctions (Figure 3a). The slices are 4.5 Å thick, corresponding to the thickness of an aromatic mesogenic side‐group, or a column stratum. Figure 3b displays the *ρ* distribution in all 12 slices cut across the double helix, showing how the two strands rotate around a common axis. The green arrow in each slice points from the *ρ* maximum of the gold strand to that of the red strand. A *ρ* maximum corresponds to the center of the “raft” of aromatic cores of the side‐groups. In the double‐helical columns, a bundle of ca. 8 PMS backbones runs through the central axis (see Figure 4a). Two rafts of ca. 4 side‐groups, each are attached to opposite sides of the bundle (Figure 3c). The raft pairs, symbolized as dumbbells in Figure 3d, rotate around their PMS axis. The aromatic mesogens (the dumbbell “weights”) combine in two separate, high*‐ρ* helical strands (Figure 3e,f). Similar to what had been proposed previously in polycatenar small molecules^[^ ^24^, ^30^, ^32^, ^34^, ^35^ ^]^ and polymers,^[^ ^38^ ^]^ a twist in the orientation of successive rafts is necessary to reduce steric clashes between the alkyl end‐tails.

From the plot in Figure 3g and the associated model in Figure 3e,f, it is evident that the twist between two successive rafts is larger close to the junction and smaller in the middle of the segment. This is expected, as the steric clash of end‐groups is larger at junctions where six strands meet. In Figure 2d, it can be seen that at the junction point (indicated by the green arrow), the red and the gold strands in each of the three adjoining double‐helices are twisted well away from each other so that the staggered strands around the junction appear as a 6‐arm star with $\bar{3}$ symmetry. In fact, in the double helical columnar phase with no junctions, formed by another polymer in the same series (**Si9‐12**), similar non‐uniform twisting of rafts along the column has been noted, resulting in alternation of splay and twist zones.^[^ ^38^ ^]^ Such behavior is consistent with the general principle that concentrating large defects (twists in this case) in small areas lowers the system's energy, as exemplified, e.g., by dislocations and disclinations.

With increasing temperature, SAXS intensities show significant changes, with the (400) and (422) peaks weakening in particular. A comparison of isoelectron surfaces enclosing the highest*‐ρ* 11% of the volume in Figure 4a shows that the two strands of the double‐helix move closer. They start to merge at 135 °C and become inseparable at 155 °C, even though the hint of supertwisting still remains. This could be explained by increased thermal fluctuations. At these higher temperatures, the boundaries between the aromatic (Ar) and the PMS + spacer (Si) moieties become more diffuse. The increased intermixing between Ar and Si is also supported by the narrowing of the gap between high (Ar) and low (Si) electron density peaks in the *ρ* histograms in Figure S9. The reduced phase separation and partial “unbundling” and expansion of the PMS backbones may also explain the broad heat capacity (*C_p_*) maximum around 120 °C–130 °C on heating (Figure 1b) as well as the simultaneous increase in lattice parameter (inset in Figure 1d). “Unbundling” the PMS backbones and the resulting expansion of the Si moiety means expansion of the narrow end of the wedge, i.e., the side‐group of **Si3‐9**. This would tend to reduce the tilt of the side‐groups, causing an increase in distance between the two gyroid networks. It would also drive the structure toward smectic and possibly explain the unusual Iso→Sm→Cubic phase sequence observed with decreasing temperature (Figures 1b,e and S6).

Compared to the typical ∼10 nm (or smaller) gyroid unit cell parameter in small polycatenar molecules,^[^ ^6^, ^7^, ^11^, ^12^, ^15^, ^16^ ^]^ the value of >14 nm measured in **Si3‐9** is considerably larger. This could be attributed to the cross‐section of the columnar segments in the polymer being effectively double that in small molecules, as it contains two instead of one strand of rafts. The larger lattice parameter also means the double‐helical network segment is longer by 40%. This may provide a possible explanation for the “supertwist” in our networks, as the increase from 70.5° to 109.5° is 55%. Our calculations (Table S6) show that on each double helical segment between two junctions there are ∼85 monomer units, organized in ∼11 strata or raft pairs, each raft containing ∼4 monomer units. There is a twist of ∼10° on average between neighboring strata. A very similar twist angle between consecutive strata is found in bicontinuous phases of small polycatenar molecules, where a stratum contains only one raft, centered on the column axis.^[^ ^30^, ^32^, ^34^, ^35^ ^]^

Another factor that could favor supertwisting over normal twisting in the 4NG phase is space filling. The alkyl end‐chains should be distributed evenly around the minimum surface and its surroundings. Due to the formation of double‐helical segments, the length ratio of the end‐chains to that of the diameter of the rigid inner part is significantly smaller; that means they will stay more closely to the position where the molecular director is projected onto the minimum surface. Therefore, it is more important in **Si3‐9** that the trajectory of the twisting molecular director projected onto the minimum surface should distribute more evenly. In Figure 4b,c, we have plotted the trajectories of molecular directors projected from the networks to the minimum surface, with normal twist (b) or supertwist (c). It turns out that for the supertwisted structure, the trajectory fluctuates between points on the minimum surface with symmetry $\bar{3}$ and $\bar{4}$, while for normal twist, they move between points with symmetry $\bar{3}$ only. The area of the individual patches bounded by trajectories on the minimum surface of the supertwisted structure is half of that of the normal one, if scaled to the same unit cell parameter. In the supertwisted structure, the end‐tails can therefore fill the gaps in between more easily. We would like to point out that the normal twisted and supertwisted double helices in one of the subspaces of the gyroid phase are the two simplest topological ways in which multiple chiral strands can be arranged around each other.^[^ ^39^ ^]^

Our results raise the interesting question as to whether the single strands in the DG phase previously found in non‐polymeric polycatenars could be supertwisted too. As mentioned above, results from resonant scattering are consistent with the normal twist model.^[^ ^31^ ^]^ However, as at the time the supertwisted model was not examined in comparison, such experimental results should be revisited in view of the new findings in this paper.

It should be noted that using a self‐consistent field theory, it has been demonstrated that in a gyroid phase formed by ABC triblock copolymer, where A block is chiral, the preferred nematic twist in the subspace of the chiral block is the reverse of that of the network subspace itself.^[^ ^40^ ^]^ Even though this is also “supertwisting”, the underlying reasons for the triblock behavior could be quite different, as the length scale of the subspace is much larger than that of the repeating unit in block copolymers, while in our system the two scales are comparable.

It is worth noting that there is a kind of Copernican revolution in molecular arrangement in the current polymer compared with that in small molecules. The rod‐like mesogens in small polycatenars occupy the center of the single‐strand columnar segment. In the polymer, it is the bundle of PMS backbones together with the alkyl spacers that occupy the center, with the mesogens shifted toward the periphery and clustered into the two helical strands. Had that inversion not happened, the backbones would have been scattered around the periphery. Clearly, the driving force for the inversion is the tendency of the PMS backbones to phase separate. This tendency is seen to wane at high temperature as strands tend to merge (Figure 4a) and disappear completely when and if the gyroid gives way to the smectic close to *T_i_*.

### Assembly in Ultra‐Thin Films and Disassembly at High Temperature

**Figure 5:**
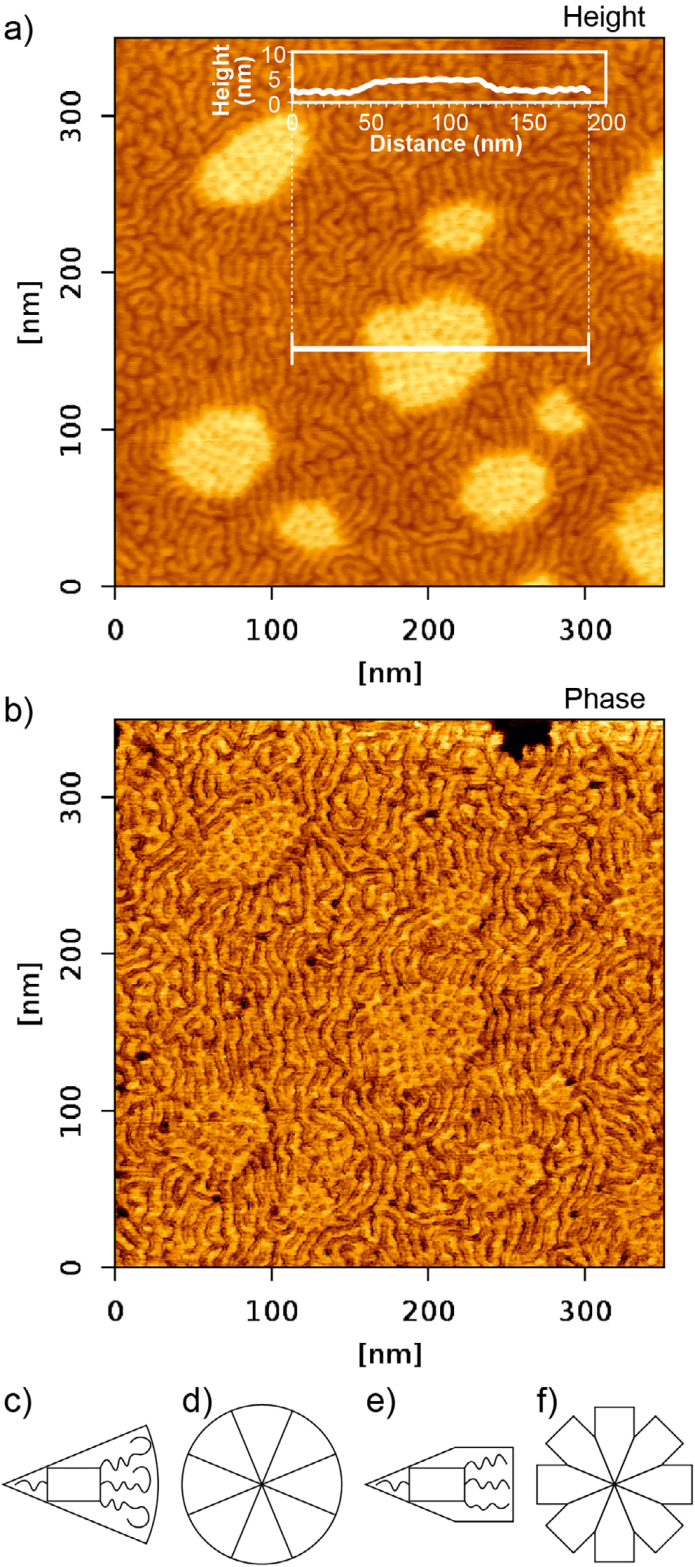
AFM height a) and phase b) images of the same area of a very thin film of **Si3‐9** prepared by drop casting, then heating to 160 °C and cooling at 0.1 °C min^−1^ from 140 °C to room temperature. Inset in a): height profile showing that the thickness of each layer is ∼2.5 nm, similar to that of the width of a single column. c) and d) Molecules with longer end‐chains adopt a wedge shape and form a columnar phase. e) The cross‐sectional area of shorter end‐chains remains constant. f) Columns with shorter chains have insufficient peripheral volume, resulting in the formation of junctions.

AFM images of an extremely thin film of **Si3‐9** (2–3 nm) are displayed in Figure 5a,b. Most of the area shows single columns containing a bundle of main chains surrounded by their side‐groups. While the resolution is insufficient to discern their internal structure, the wiggly column trajectory suggests high flexibility. This is understandable, as the end‐chains do not provide sufficient volume to rigidify and straighten the columns.^[^ ^41^ ^]^ Extending the end‐chains makes them more likely to fold back, increasing their effective cross‐section area and filling the space at the column periphery,^[^ ^42^ ^]^ see Figure 5c,d. In contrast, columns with shorter chains need junctions to make up for their insufficient peripheral volume, resulting in the gyroid cubic (Figure 5e,f). Thus, polymers **Si3‐12** ^[^ ^37^ ^]^ and **Si9‐12**,^[^ ^38^ ^]^ form the columnar phase, while **Si3‐9** forms the gyroid. In the gyroid, end‐chains are squeezed out of the “armpit” of the junction, allowing them to cluster together and fill the space between the junctions.

Notably, in the AFM image, there are also a few patches seen as bright in the height image in Figure 5a. There, the film has double thickness, and a second layer of columns is seen, most clearly in the phase image, Figure 5b. In the second layer, the columns are roughly perpendicular to those below, and in both layers, they appear to be straight. Whether or not the structure of the bilayer is exactly as in the gyroid, the observed straightening of the columns is consistent with the formation of junctions where columns cross. As always in bicontinuous phases, junctions serve to mitigate the shortfall in volume afforded by the end‐tails when their size is insufficient to support a columnar phase.

Generally, an important piece of information provided by the AFM images is that self‐assembly of columns around a bundle of main chains is self‐sufficient and does not rely on a higher hierarchical level of order on a 2D or 3D lattice.

**Figure 6:**
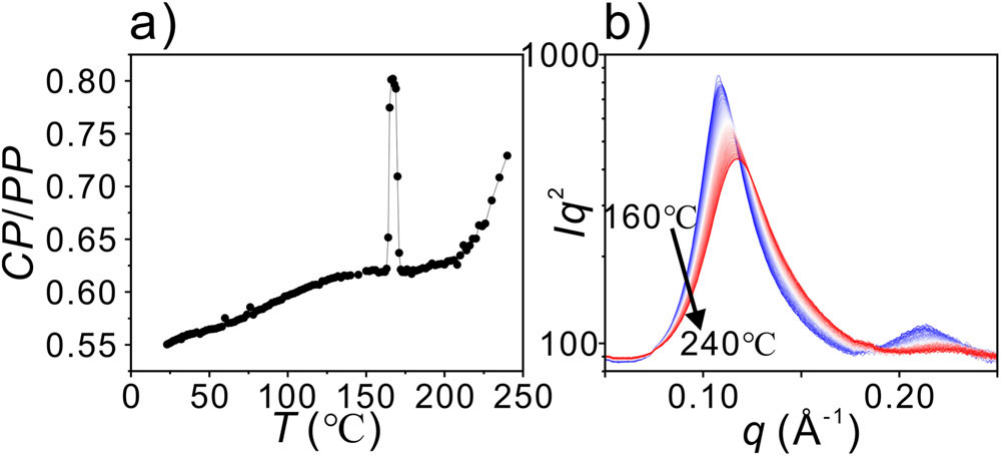
a) DF ratio CP/PP of **Si3‐9** versus temperature. Note that the sharp peak around 166 °C is an artifact due to the brief appearance of the birefringent smectic phase before isotropization. As mentioned further above, only an extremely slowly cooled gyroid of high perfection melts directly to Iso liquid, and the sample used here had been cooled at a higher rate. b) Powder SAXS profile of Iso melt with increasing temperature above the transition from the gyroid.

Local assembly also shows a degree of independence from the long‐range LC structure at the high‐temperature end. We used depolarized fluorescence (DF) as a measure of molecular mobility. The sample was irradiated with polarized exciting light, and the ratio of intensities of cross‐polarized (CP) and parallel‐polarized (PP) fluorescence was measured with increasing temperature. The higher the mobility, the more fluorophores reorient during the excitation lifetime; hence, CP/PP is higher. Figure 6a shows a slow increase in mobility of **Si3‐9** between room *T* and 206 °C, notably until 40 K above the transition to isotropic liquid. Only above this temperature does mobility start to increase steeply, indicating the start of true disassembly of local aggregates. Figure 6b shows that a significant change in the SAXS curve of the melt starts only well above *T_i_*, i.e., above 200 °C (white–red regions), but not much below it (blue region). Particularly telling is the loss of the second SAXS maximum that happens above ca. 200 °C, which indicates the undoing of the double helices.

Both the gradual but steep increase in mobility (Figure 6a) and broadening of the main SAXS peak and loss of the second (Figure 6b) are consistent with findings of a recent study that showed how most complex LC phases, including gyroid and smectic, are gradually and reversibly pre‐assembled in the melt, with only a minor entropic drop at the phase transition to the long‐range ordered LC phase.^[^ ^43^ ^]^ In small molecules, this is usually accompanied by a pronounced peak in heat capacity (*C_p_*), which can be very broad and hard to identify in polymers, as is the case in **Si3‐9**. However, continuous transitions, with *C_p_* humps, could also occur within the temperature range of an LC phase, as is the case in the current polymer around 120 °C–130 °C (see Figure 1b, inset in 1d, and associated discussion above).

## Conclusion

A new subclass of gyroid cubic LC is discovered in a polymer with polycatenar side groups. The two antichiral gyroid networks consist of two aromatic strands each. In this 4NG, the first of its kind, two strands form a double helix that “supertwists” by ±109.5° between successive network junctions, while the dihedral angle between junctions is ∓70.5°. Previously the assumption was that the twist of the molecular rafts follows the 70.5° twist of the network channel, but this could not be confirmed directly on single strands. A possible reason for the opposite chiralities of the double helix and the network is the large unit cell size and the associated 40% larger than normal distance between junctions. A more even distribution of flexible end‐chains also favors supertwisting. In addition, thin‐film AFM demonstrates that columnar strands containing bundled polymer chains exist even when gyroid formation is prevented by confinement. Furthermore, DF shows that the system's mobility does not increase at the transition from LC to liquid, but that the increase in mobility starts in earnest only 40 K above the transition, indicating that serious molecular disassembly starts only well into the liquid state.

## Supporting Information

The authors have cited additional references within the Supporting Information.^[^ ^44^, ^45^ ^]^

## Conflict of Interests

The authors declare no conflict of interest.

## Supporting information



Supporting information

## Data Availability

The data that support the findings of this study are available from the corresponding author upon reasonable request.
