# Supplementary material for: Gyroid Labyrinth of Supertwisted Double Helices in a Liquid Crystal Polymer
Source: Angew Chem Int Ed Engl. 2025 Dec 4;65(4):e22314. doi: 10.1002/anie.202522314 (PMC12828463; doi:10.1002/anie.202522314)
Supplement: Supplementary file 1 — Supporting information [file ANIE-65-e22314-s001.docx]

**Gyroid Labyrinth of Supertwisted Double Helices in a Liquid Crystal Polymer**

Yumin Tang,^[a]^ Yi-nan Xue,^[b]^ Shu-Gui Yang,^[b]^ Ruibin Zhang, ^[a]^ Feng Liu,^[b]^ Xiangbing Zeng,*^[a]^ Goran Ungar*^[a,b]^

[a] School of Chemical, Materials and Biological Engineering, University of Sheffield, Sheffield S1 3JD, UK

*Email : [x.zeng@sheffield.ac.uk](mailto:x.zeng@sheffield.ac.uk)

[b] Shaanxi International Research Center for Soft Matter, School of Material Science and Engineering, Xi'an Jiaotong University, Xi’an 710049, China

*Email: g.ungar@xjtu.edu.cn

**Table of Contents**

[**1.** **Methods of characterization** 2](#_Toc213859021)

[**1.1.** **Characterization of chemical structure** 2](#_Toc213859022)

[**1.2.** **Polarized optical microscopy** 2](#_Toc213859023)

[**1.3.** **Differential scanning calorimetry** 3](#_Toc213859024)

[**1.4.** **Synchrotron small/wide angle *X*-ray scattering (*SAXS*/*WAXS*)** 3](#_Toc213859025)

[**1.5.** **Grazing incidence small angle X-ray scattering experiments (*GISAXS*）** 4](#_Toc213859026)

[**1.6.** **Electron density map reconstruction** 4](#_Toc213859027)

[**1.7.** **Atomic Force Microscopy** 4](#_Toc213859028)

[**1.8.** **Fluorescence depolarization measurement** 4](#_Toc213859029)

[**2.** **Synthesis and analytical data** 5](#_Toc213859030)

[**2.1.** **Abbreviations** 5](#_Toc213859031)

[**2.2.** **Chemicals and Materials** 5](#_Toc213859032)

[**2.3.** **Synthesis of Compounds** 5](#_Toc213859033)

[**2.3.1.** **Synthesis of**  **3,4,5-tris(nonyloxy)benzoic acid(compound 4)** 6](#_Toc213859034)

[**2.3.2.** **Synthesis of 4-(allyloxy) benzoic acid (compound 8)** 7](#_Toc213859035)

[**2.3.3.** **Synthesis of 4'-hydroxy-[1,1'-biphenyl]-4-yl 4-(allyloxy)benzoate (compound 10)** 7](#_Toc213859036)

[**2.3.4.** **Synthesis of 4'-((4-(allyloxy)benzoyl)oxy)- [1,1'-biphenyl]-4-yl 3,4,5-tris(nonyloxy)benzoate compound (Vin 3-9)** 8](#_Toc213859037)

[**2.3.5.** **Synthesis polymer Si3-9** 8](#_Toc213859038)

[**3.** **DSC** 12](#_Toc213859039)

[**4.** **POM** 13](#_Toc213859040)

[**5.** **Powder *SAXS*/*WAXS*** 14](#_Toc213859041)

[**6.** **Electron density map reconstruction** 17](#_Toc213859042)

[**7.** **Selection of phase combinations in the reconstruction of electron density maps** 19](#_Toc213859043)

**References**………………………………………………………………………………………………………………………………………21

**Section S1**

1. **Methods of characterization**
   1. **Characterization of chemical structure**

^1^H-NMR spectra (600 MHz), ^13^C-NMR spectra (151 MHz) were recorded on a Bruker Advance III HD 600 spectrometer using chloroform-*d* (CDCl_3_) and dimethyl sulfoxide-*d*_6_ (DMSO-*d*_6_) as solvents and tetramethylsilane (TMS) as internal standard at 298K. Coupling constants (J) are denoted in Hz and chemical shifts (δ) in ppm. Multiplicities are denoted as follows: br = broad, s = singlet, d = doublet, m =multiplet, t = triplet, q = quartet, p = pentet, dt = doublet of triplets, dq = doublet of quartets, dd = doublet of doublets, ddd = doublet of doublets of doublets, ddt = doublet of doublets of triplets. High resolution mass spectral (HRMS) data were obtained on an electrospray (ESI) mass time-of-flight spectrometer (Waters I-Class VIONIMS QToF). The gel permeation chromatographic (GPC) analysis for compound **Si3-9** was performed on a Waters 1515-2707-2414 instrument, solvent CHCl_3_, flow rate 1mL/min, temperature 35°C, using polystyrene standards. GPC analysis for poly(methylhydrosiloxane) trimethylsilyl terminated (PMHS) was carried out on an Agilent 1260 Infinity II instrument, solvent CHCl_3_, flow rate 0.6 mL/min, temperature 50°C, with polymethyl methacrylate as standard.

- 1. **Polarized optical microscopy**

Optical micrographs of mesophases with crossed polarizers were recorded on an Olympus BX-51 microscope equipped with a Linkam LTS420 hot stage and a T95-HS controller. The sample was heated to isotropic melt between glass slides and cooled to desired temperature at a rate of 1 K/min.

- 1. **Differential scanning calorimetry**

DSC thermograms were recorded on a TA Instruments DSC250 at the heating/cooling rates of 5 K·min-1. The samples were dried in vacuo at room temperature for 2 days prior to the scan, and the DSC cell was flushed with dry nitrogen. Sapphire was used to calibrate Tzero and Indium was used to calibrate the temperature and heat flow. Aluminum sample pans were used.

- 1. **Synchrotron small/wide angle *X*-ray scattering (*SAXS*/*WAXS*)**

*SAXS*/*WAXS* experiments were carried out at beamline BL16B1 at Shanghai Synchrotron Radiation Facility (SSRF), and at beamline I22 Diamond Light Source (DLS), U.K., and at beamline BM28 European Synchrotron Radiation Facility (ESRF), France. Pilatus 2M detector was used to record *SAXS*/*WAXS* patterns at I22. MAR-165 CCD camera was used at BM28. Sample was held in capillary of 1.0 mm in diameter. A Linkam hot stage with a thermal stability within 0.2 ^o^C was used, with a hole for the capillary drilled through the silver heating block and mica windows attached to it on each side. Capillary was rotated by a DC motor at ca. 100 rpm during recording to improve signal averaging and to reduce radiation damage. Temperature and *q-*vector value calibrations as well as linearization were performed by using several orders of layered reflections from silver behenate and a series of *n*-alkanes.

- 1. **Grazing incidence small angle X-ray scattering experiments (*GISAXS*）**

*GISAXS* experiments were conducted at beamlines I16 DLS, U.K. The GISAXS pattern was recorded by using a Pilatus 2M detector. Thermal treatment on sample was enabled with a customised thermal setup supervised by a Eurotherm temperature controller. Sample was prepared onto a silicon wafer. *n*-tetracontane was used to calibrate the sample to detector distance as well as the real temperature, from the known melting point of the standard.

- 1. **Electron density map reconstruction**

The electron density map ($\rho$-map) was reconstructed according the established procedure as follows.^^[[1]](#endnote-1)^^ In principle, the scattering intensity differs for different areas of a molecule in the *X*-ray scattering/diffraction. Electron density distribution ($\rho\left( x,y,z \right)$) in a unit cell is known to be related to the structure factor ($F\left( hkl \right)$) by the inverse Fourier transform. This relationship could be described as:

$\rho\left( x,y,z \right)=\frac{1}{V}\sum_{hkl} F\left( hkl \right)e^{\left( -2\pi i\left( hx+ky+lz \right) \right)}$

Giving the relationship between $F\left( hkl \right)$ and $I\left( hkl \right)$ to be:

$$F\left( hkl \right)=\frac{1}{const.}\sqrt{I\left( hkl \right)}$$

Electron density distribution could be further formulated in general as:

$\rho\left( x,y,z \right)=\frac{1}{const.}\sum_{hkl} \sqrt{I\left( hkl \right)}e^{(-2\pi i\left( hx+ky+lz \right)+i\phi_{hkl})}$.

Here, the $\rho\left( x,y,z \right)$ means the electron density distribution, $V$ is the volume of unit cell, the $I\left( hkl \right)$ represents the intensity of the *X*-ray scattering/diffraction peak, and the $\phi_{hkl}$ is the phase angle of the $F\left( hkl \right)$ of the $(hkl)$ reflection. Generally, the phase angle should be $0\sim2\pi$, as the $F(hkl)$ is a complex number. While, for structure with centro-symmetric space group, $F(hkl)$ turns out to be a real value and the phase angle to be either 0 or $\pi$. These conditions will simplify the calculation.

- 1. **Atomic Force Microscopy**

Tapping-mode atomic force microscopy (AFM) measurements were conducted at room temperature using a JPK BioAFM system (Bruker, Germany). Aluminium-coated silicon probes with tip diameters of approximately 7–10 nm were employed.

- 1. **Fluorescence depolarization measurement**

Fluorescence depolarization was measured using the same Olympus microscope with a Linkam hot stage, as described above. A Thorlabs’ M365LP1 Mounted LED with a wavelength of 365 nm was passed through a polarizer, a BP 340-390 excitation filter, a DM 410 dichroic mirror, before reaching the sample. Fluorescence was recorded in reflection mode, after passing through the dichroic mirror, a LP420 emission filter, and an analyzer that was either parallel (𝐼*_∥_*) or perpendicular (𝐼_⊥_) to the polarization direction of exciting light, respectively.

1. **Synthesis and analytical data**
   1. **Abbreviations**

PMHS - polymethylhydrosiloxane (trimethylsilyl terminated)

DCM - dichloromethane

DMF - dimethylformamide

DMAP - 4-dimethylaminopyridine

EDC·HCl - 1-ethyl-3-(3-dimethylaminopropyl)carbodiimide hydrochloride

EtOAc - ethyl acetate

PE - petroleum ether (fraction 60-90)

THF – tetrahydrofuran

MeCN - acetonitrile

CHCl3 – chloroform TLC – thin layer chromatography

- 1. **Chemicals and Materials**

Methyl 3,4,5-trihydroxybenzoate (methyl gallate, 98%, InnoChem), 1- Bromononane (98.5%, InnoChem), methyl 4-hydroxybenzoate (99%), (InnoChem) 4,4’-dihydroxybiphenyl (98%), (InnoChem), polymethylhydrosiloxane (trimethylsilyl terminated, Mn,GPC=3.2 kg mol-1, PDI = 2.43) from Gelest. Dry THF and dry DMF were ordered from Beijing InnoChem Science & Technology Co., Ltd. Dichloromethane (DCM) was dried by reflux for at least 12 hours with calcium hydride and stored with activated standard 3A grade molecular sieve. All other chemicals and solvents were of analytical grade and were used as received. Column chromatography was performed with silica gel (300-400 mesh) produced by Qingdao Ocean Chemical Co., Ltd. Reactions requiring an inert gas atmosphere were conducted under nitrogen.

- 1. **Synthesis of Compounds**

The **Vin3-9** compound and the **Si3-9** polymer are new and they were prepared according to the literature.^^[[2]](#endnote-2)^,^[[3]](#endnote-3)^^

^^

**Scheme S1.** Reagents and conditions for synthesis of compounds: *i*) K_2_CO_3_, dry DMF, 80 ^o^C, 24 hrs; *ii*) KOH, ethyl alcohol, 80 °C, 12 hrs; *iii*) K_2_CO_3_, acetone, 80 °C, 24 hrs; *iv*) DMAP, EDC^.^HCl, dry DMF, 0 ^o^C for 2 hrs and then 48 hrs at r.t.; *v*) DMAP, EDC∙HCl, dry DCM, 0 ^o^C for 2 hrs and then 48 hrs at r.t.; *vi*) Karstedt’s catalyst, 60^o^C, 48 hrs, dry toluene.

- - 1. **Synthesis of**  **3,4,5-tris(nonyloxy)benzoic acid(compound 4)**

*Step i - Williamson Etherification Reaction:* Methyl 3,4,5-trihydroxybenzoate (9.2 g, 50.0 mmol, 1 eq.) and anhydrous K2CO3 (27.6 g, 200.0 mmol, 4 eq.) were purged with nitrogen. A dry DMF (100 mL) and 1-Bromononane (51.8 g, 250 mmol, 5 eq.) were added and the solution was refluxed for 16 hours. The DMF was removed and the organic phase was extracted with EtOAc (3x 50 mL). The collected organic layers was washed with H2O (2x100 mL) and dried over anhydrous Mg_2_SO_4_, filtered and concentrated. The crude product was purified by column chromatography (PE/EtOAc = 5/1, TLC: Rf = 0.5). It was obtained 12 compound **3** as pale yellow oil (27.9 g, η = 99 %).

*Step ii - Hydrolysis Reaction:* A mixture of compound 3 (27.9 g, 49.5 mmol, 1 eq.) dissolved in MeOH (220 mL) and KOH (11.2 g, 200.0 mmol, 4 eq.) dissolved in H_2_O (32 mL). was allowed to react at 80 ^o^C for 12 hours. It was then cooled to room temperature, followed by the addition of an equal amount of ice/water. The pH of the solution was adjusted stepwise with HCl until pH = 2-3 until a solid stop to precipitate. Recrystallization of precipitate gave the compound 4, which was dried in the oven for 48 hours. (25.7g, η = 95 %).

**Compound 4: 1H NMR** (600 MHz, CDCl_3_) δ 7.33 (s, 2H, Ar-*H*), 4.04 (dq, *J* = 13.8, 7.1 Hz, 6H, OC*H*_2_), 1.82 (p, *J* = 7.4 Hz, 4H, OCH_2_C*H*_2_), 1.76 (p, *J* = 7.4 Hz, 2H, OCH_2_C*H*_2_), 1.48 (p, *J* = 7.7 Hz, 6H, OCH_2_CH_2_C*H*_2_), 1.32 (m, 30H, C*H*_2_), 0.89 (q, *J* = 7.5 Hz, 9H, C*H*_3_).

- - 1. **Synthesis of 4-(allyloxy) benzoic acid (compound 8)**

*Step iii - Williamson Etherification Reaction:* The methyl 4-hydroxybenzoate (7.6 g, 50.0 mmol, 1 eq.) and anhydrous K_2_CO_3_ (27.6 g, 200.0 mmol, 4 eq.) were purged with nitrogen. Acetone (250 mL) and allyl bromide 6.7g, 55 mmol, 1.1 eq.) were added and the mixture was refluxed for 16 hours. The organic phase was extracted with EtOAc (3x100 mL). The collected organic layers washed with H_2_O (2x100 mL) and dried over anhydrous Mg_2_SO4, filtered and concentrated. The crude product was purified by column chromatography (PE/DCM = 1/4, TLC: Rf = 0.6). It was obtained compound 7 as pale yellow oil (9.4 g, η = 98 %).

*Step ii – Hydrolysis Reaction:* A mixture of compound 7 (7.4 g, 38.5 mmol, 1 eq.) dissolved in MeOH (200 mL) and KOH (10.8 g, 193 mmol, 5 eq.) dissolved in H_2_O (53 mL) was allowed to reacted at 80 ^o^C for 12 hours. It was then cooled to room temperature, followed by the addition of an equal amount of ice/water. The pH of the solution was adjusted stepwise with HCl until pH = 2-3 when a solid began to precipitate. Recrystallization of precipitate gave the compound 8 as white solid, which was dried in the oven for 48 hours. (6.2 g, η = 90 %).

**Compound 8**: **^1^H NMR** (600 MHz, CDCl_3_) δ 8.28 – 7.84 (m, 2H, Ar-*H*), 6.96 (dd, *J* = 6.7, 4.9 Hz, 2H, Ar-*H*), 6.06 (ddt, *J* = 17.2, 10.6, 5.3 Hz, 1H, =C*H*), 5.44 (dd, *J* = 17.3, 1.4 Hz, 1H, =C*H_a_*H_b_), 5.33 (dd, *J* = 10.5, 1.3 Hz, 1H, =CH_a_*H_b_*), 4.78 – 4.43 (m, 2H, C*H_2_*).

- - 1. **Synthesis of 4'-hydroxy-[1,1'-biphenyl]-4-yl 4-(allyloxy)benzoate (compound 10)**

*Step iv – Esterification Reaction:* A solution of compound 8 (5.9 g, 33.0 mmol, 1 eq.), compound 9 ( 30.7 g, 165.0 mmol, 5 eq.), EDC·HCl (9.5 g, 49.5 mmol, 1.5 eq.), DMAP ( 2.0 g, 16.5 mmol, 0.5 eq.) in dry DMF (200 mL) was stirred at 0 ^o^C, under nitrogen. It was then allowed to warm to room temperature and stirred for an additional 24 hours. The organic phase was extracted with EtOAc.

The volume of EtOAc was removed on the rotary evaporator. The crude product was recrystallized from ethanol (3x 100 mL) and then was purified on column chromatography (DCM/EtOAc = 10/1, TLC: Rf = 0.7). A white solid was obtained (5.4 g, η = 47 %).

**Compound 10: ^1^H NMR** (600 MHz, DMSO-*d*_6_) δ 9.58 (s, 1H, O*H*), 8.09 (d, *J* = 8.9 Hz, 2H, Ar-*H*), 7.64 (d, *J* = 8.6 Hz, 2H, Ar-*H*), 7.51 (d, *J* = 8.6 Hz, 2H, Ar-*H*), 7.29 (d, *J* = 8.6 Hz, 2H, Ar-*H*), 7.15 (d, *J* = 8.9 Hz, 2H, Ar-*H*), 6.86 (d, *J* = 8.6 Hz, 2H, Ar-*H*), 6.08 (ddd, *J* = 22.5, 10.5, 5.2 Hz, 1H, =C*H*), 5.44 (dd, *J* = 17.3, 1.6 Hz, 1H, =C*H_a_*H_b_), 5.31 (dd, *J* = 10.6, 1.4 Hz, 1H, =CH_a_*H_b_*), 4.71 (d, *J* = 5.2 Hz, 2H, =CHC*H_2_*).

- - 1. **Synthesis of 4'-((4-(allyloxy)benzoyl)oxy)- [1,1'-biphenyl]-4-yl 3,4,5-tris(nonyloxy)benzoate compound (Vin 3-9)**

*Step v – Esterification Reaction:* Compound **4** (2.8 g, 5.0 mmol, 1 eq.), compound 10 (1.7 g, 5.0 mmol, 1 eq.), DMAP (1.5 eq.) and EDC·HCl (1.5 eq.) were dissolved in dry DCM (200 mL). The mixture was stirred 48 hours at room temperature and then extracted with DCM. The solvent was removed and the crude product was purified by column chromatography (PE/DCM = 1/2, TLC: Rf = 0.5). It was obtained an white solid (3.8 g, η = 87 %).

**Compound Vin3-9: ^1^H NMR** (600 MHz, CDCl_3_) δ 8.18 (d, *J* = 8.3 Hz, 2H), 7.63 (d, *J* = 8.1 Hz, 4H), 7.43 (s, 2H), 7.28 (t, *J* = 7.8 Hz, 4H), 7.01 (d, *J* = 8.3 Hz, 2H), 6.08 (ddt, *J* = 16.4, 10.7, 5.6 Hz, 1H), 5.46 (d, *J* = 17.3 Hz, 1H), 5.35 (d, *J* = 10.5 Hz, 1H), 4.64 (d, *J* = 5.3 Hz, 2H), 4.07 (q, *J* = 6.3 Hz, 6H), 1.84 (p, *J* = 6.9 Hz, 4H), 1.77 (p, *J* = 6.9 Hz, 2H), 1.49 (p, *J* = 7.5 Hz, 6H), 1.42 – 1.20 (m, 30H), 0.88 (t, *J* = 5.8 Hz, 9H). **^13^C NMR** (151 MHz, CDCl_3_) δ 165.1, 164.9 (*C*=O), 163.0, 153.0, 150.6, 150.5, 143.0 (*C_Ar_*-O), 138.2, 138.1, 138.1 (*C_Ar_-C_Ar_*), 132.5 (*C*H=), 132.3, 128.2 (*C_Ar_*-H), 123.9 (*C_Ar_*-COO), 122.2 (*C_Ar_*-H), 122.1 (*C_Ar_-*COO), 121.9 (*C_Ar_*-H), 118.3 (*C*H_2_=), 114.6, 108.6 (*C_Ar_*-H), 73.6, 69.3, 69.0 (O*C*H_2_), 32.0, 31.9, 30.4, 29.7, 29.6, 29.6, 29.4, 29.4, 29.3, 26.1, 26.1, 22.7, 22.7 (*C*H_2_), 14.1 (*C*H_3_). HRMS (ESI, MeCN): calc. for [M+H] ^+^ (*m/z)*: 877.5613; Found: 877.5603.

- - 1. **Synthesis polymer Si3-9**

*Step vi - Hydrosilylation Reaction:* The compound VinA 3-9 (1.32 g, 1.1 eq.), and polymethylhydrosiloxane (PMHS Mn=3200 g·mol-1, PDI = 2.43) (90 uL, 1 eq.) was dissolved in dry toluene (5 mL), and then several drops of Karstedt’s catalyst were added. The reaction was purged with nitrogen and stirred at 60 ^o^C for 48 hours. The solution was cooled and precipitated with methanol. The precipitate was filtered, dissolved in DCM, and trace of catalyst was removed by passing the solution twice over Al_2_O_3_. The solution was concentrating until 10 mL and the crude product was purified by two successive recrystallization DCM/MeOH and other two recrystallization from DCM/acetone at -10 ^o^C. The product was filtered, collected, and dried under vacuum to give a white solid (0.650 g, η = 54%) Polymer **Si3-9**: Form GPC was obtained: Mn= 34.6 kg/mol, Mw=52.5 kg/mol, PDI = 1.51.

**Si3-9:**

**^1^H NMR** (600 MHz, CDCl_3_) δ 8.08 (s, 2H Ar-*H*), 7.54 (s, 4H, Ar-*H*), 7.37 (s, 2H, Ar-*H*), 7.19 (s, 4H, Ar-*H*), 6.88 (s, 2H, Ar-*H*), 4.01 (d, *J* = 22.5 Hz, 8H, OC*H_2_*), 1.92 (s, 2H, OCH_2_C*H_2_*), 1.80 (s, 6H, OCH_2_C*H_2_*), 1.55 – 1.40 (m, 6H, OCH_2_CH_2_C*H_2_*), 1.40 - 1.15 (m, 30H, C*H_2_*), 0.87 (m, 9H, C*H_3_*), 0.76 (s, 2H, SiC*H_2_*), 0.18 (d, *J* = 74.7 Hz, 4H, SiC*H_3_*).

**^13^C N****MR** (151 MHz, CDCl_3_) δ 164.9, 164.7 (*C*=O), 163.2, 152.9, 150.5, 143.0 (*C_Ar_*-O), 137.9 (*C_Ar_*-*C_Ar_*), 132.3, 128.1 (*C_Ar_*-H), 123.8 (*C_Ar_*-COO), 122.1 (*C_Ar_*-H), 119.7 (*C_Ar_*-COO), 114.2, 108.5 (*C_Ar_*-H), 73.6, 70.3, 69.2 (O*C*H_2_), 32.0, 31.9, 30.4, 29.7, 29.6, 29.5, 29.4, 29.4, 29.3, 26.1, 26.1, 23.0, 22.7, 22.7, 22.6 (*C*H_2_), 18.1, 16.6, 14.1 (*C*H_3_), 13.6 (Si*C*H_2_), 2.0, 1.0 (Si*C*H_3_).


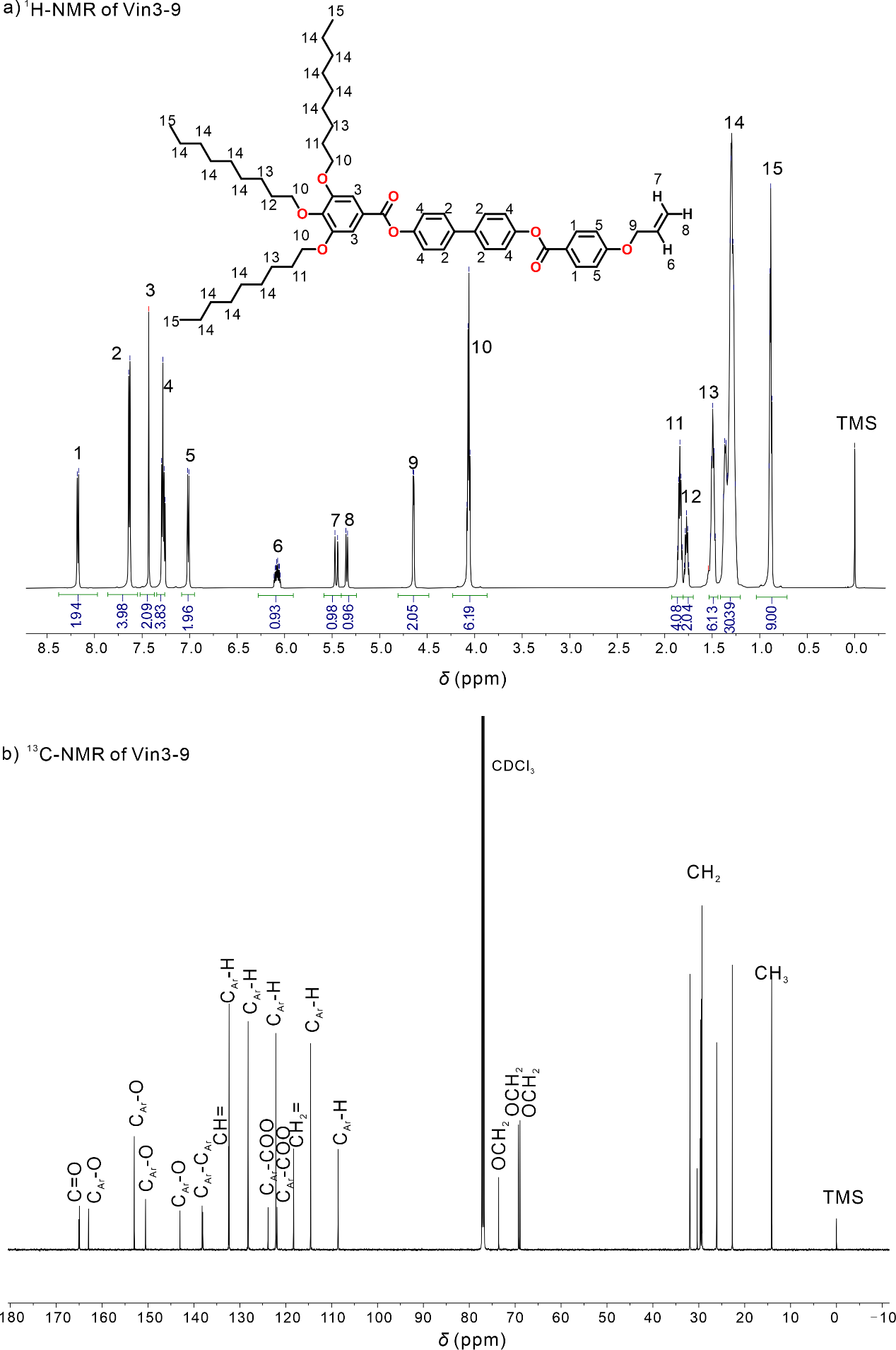


**Figure S1.** NMR spectra of **Vin3-9** compound in CDCl_3_, 298K: **a)** ^1^H-NMR (600 MHz) and **b)** ^13^C-NMR (151 MHz)


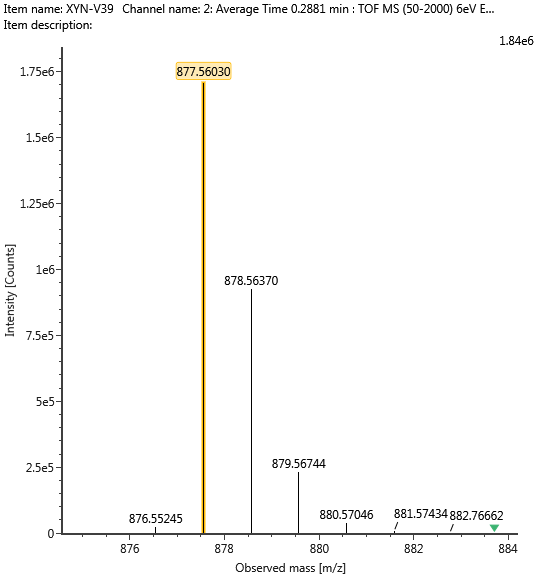


**Figure S2.** HR-MS spectrum of **Vin3-9** compound


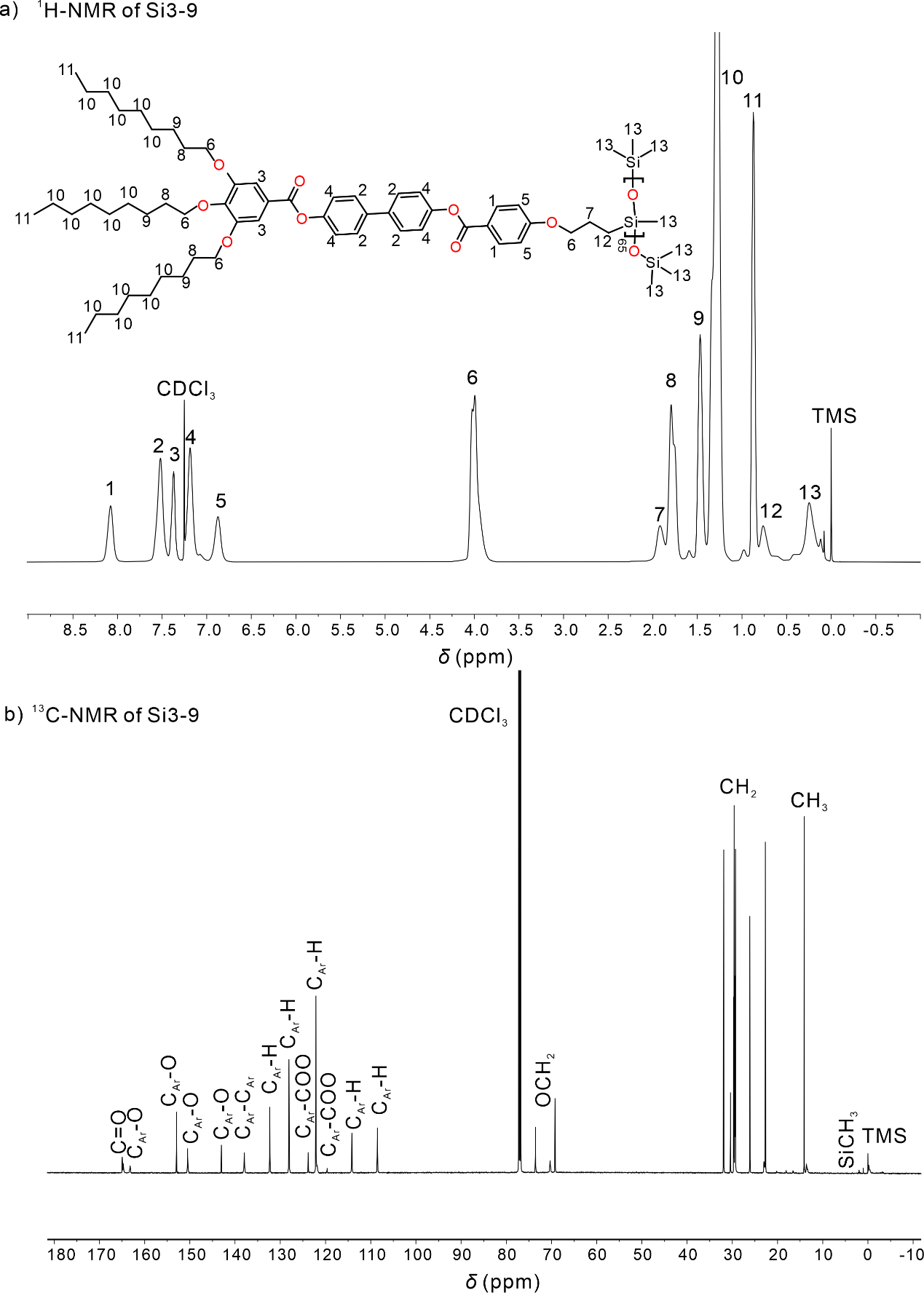


**Figure S3.** NMR Spectra of **Si3-9** polymer in CDCl_3_, 298K**: a)** ^1^H-NMR (600 MHz) and **b)** ^13^C-NMR (151 MHz)

**Section S2**

1. **DSC**


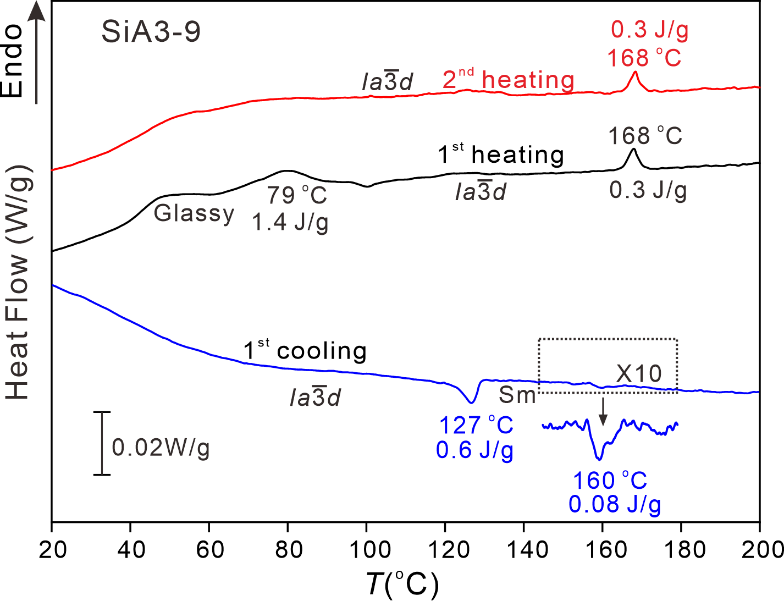


**Figure S4.** DSC thermogram of **Si3-9**. Top: red line is 1^st^ heating, black line is 2^nd^ heating of an as-dried sample; bottom: blue line is cooling. Heat/cool rates were 5 K/min. The **4NG** phase is marked with its space group $Ia\bar{3}d$.

1. **POM**


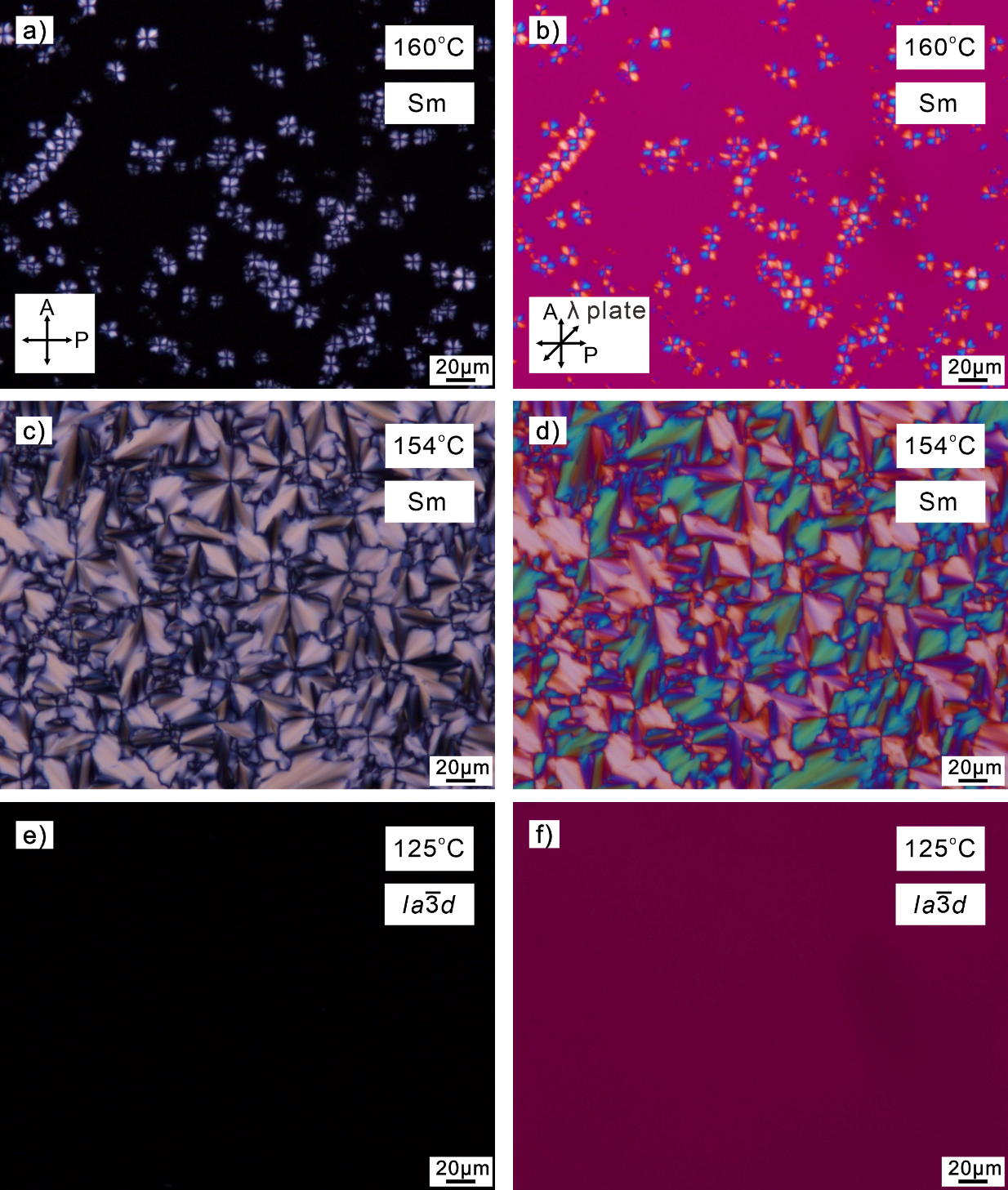


**Figure S5.** Textures of compound **Si3-9** observed between crossed polarizers after cooling from isotropic melt at 1 K/min to (a,b) 160 ^o^C, *Sm* phase, (c,d) 154 ^o^C, *Sm* phase, (e,f) 125 ^o^C, **4NG** phase , (b,d,f) with the lambda plate.

1. **Powder *SAXS*/*WAXS***


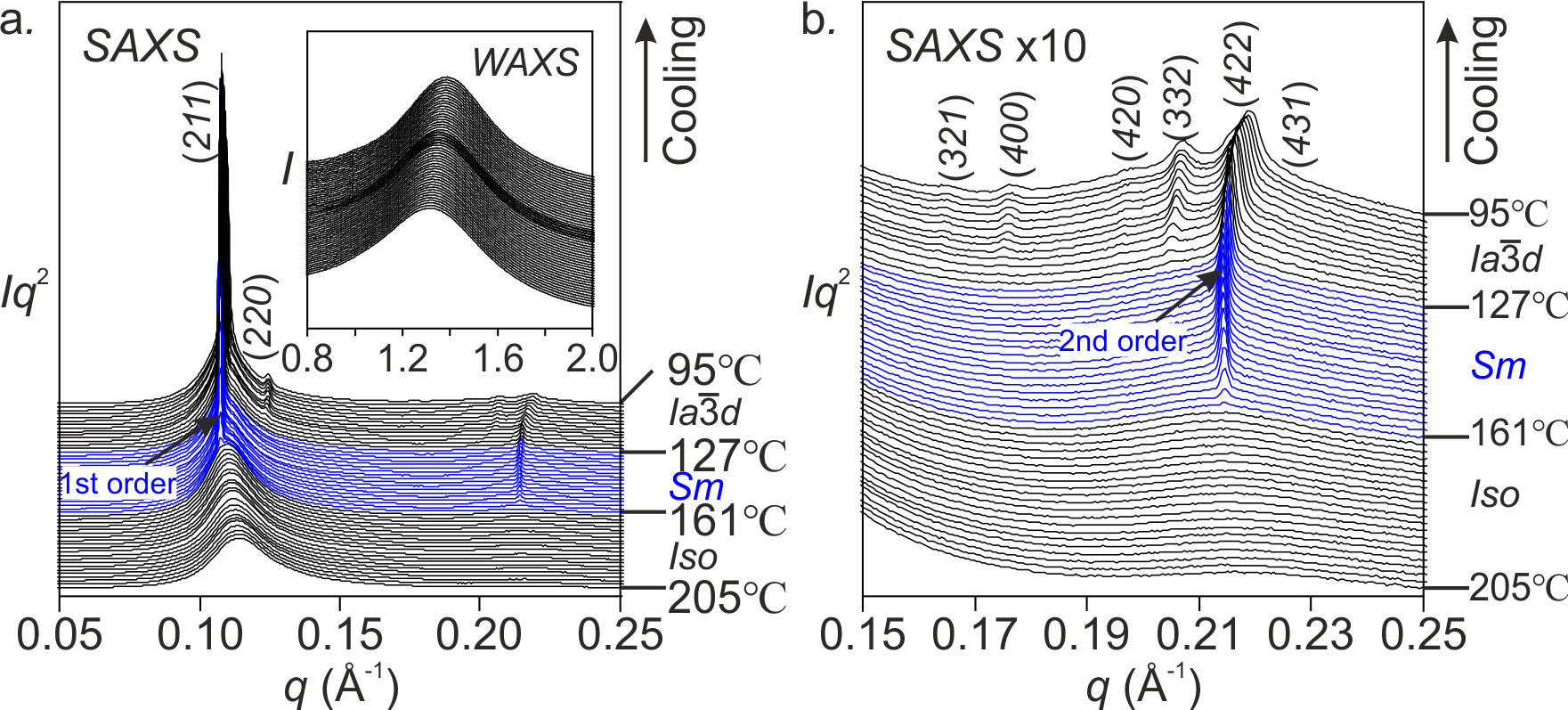


**Figure S6.** **a.** *SAXS*/*WAXS* diffractograms of the *Cub*_bi_/*Ia*$\overline{3}$*d* phase formed in **Si3-9**, recorded on 1^st^ cooling at 10 ^o^C/min from 205 ^o^C (*Iso*) to 95 ^o^C (**4NG**, space group *Ia*$\overline{3}$*d*), with Miller indices marked on the observed peaks. Diffractograms are displayed every 2 ^o^C. **b.** The corresponding magnified *SAXS* region is shown to view clearly the weak high-order peaks.

**Table S1**. Index, experimental and calculated *d*-spacing, intensity (Lorentz and Multiplicity corrected, and normalised to the strongest (*211*) peak), and the corresponding calculated lattice parameters of the **4NG** phase formed in **Si3-9** (fresh sample as received) at 95 ℃ on heating from r.t. (**4NG**) to 205 ℃ (*Iso*). The phase combination is used to reconstruct the electron density map ($\rho$-map) of the **4NG** phase.

| Phase: **4NG**, space group *Ia*$\overline{3}$*d* | | | | | |
| --- | --- | --- | --- | --- | --- |
| (*hkl*) | *d*_exp._ (Å) | *d*_calc._ (Å) | Intensity | Multiplicity | Phase |
| (*211*) | 57.8 | 57.8 | 100.00 | 24 | $\pi$ |
| (*220*) | 50.1 | 50.1 | 27.59 | 12 | $\pi$ |
| (*321*) | 37.8 | 37.8 | 0.28 | 48 | 0 |
| (*400*) | 35.4 | 35.4 | 6.78 | 6 | 0 |
| (*420*) | 31.6 | 31.6 | 0.60 | 24 | 0 |
| (*332*) | 30.2 | 30.2 | 4.21 | 24 | $\pi$ |
| (*422*) | 28.9 | 28.9 | 3.97 | 24 | $\pi$ |
| (*431*) | 27.8 | 27.8 | 0.17 | 48 | $\pi$ |
| (*521*) | 25.8 | 25.8 | 0.02 | 48 | $\pi$ |
| (*440*) | 25.0 | 25.0 | 0.26 | 12 | 0 |
| (*611*) | 22.9 | 22.9 | 0.15 | 24 | $\pi$ |
| (*543*) | 20.0 | 20.0 | 0.06 | 48 | 0 |
| (*732*) | 18.0 | 18.0 | 0.02 | 48 | $\pi$ |
| Lattice parameter: *a* = 141.6 Å | | | | | |

**Table S2**. Index, experimental and calculated *d*-spacing, intensity (Lorentz and Multiplicity corrected, and normalised to that of the (*211*) peak at 95 ℃ for comparison), and the corresponding calculated lattice parameters of the **4NG** phase formed in **Si3-9** (fresh sample as received) at 115 ℃ on heating from r.t. (**4NG**) to 205 ℃ (*Iso*). The phase combination is used to reconstruct the electron density map ($\rho$-map) of the phase.

| Phase: **4NG**, space group *Ia*$\overline{3}$*d* | | | | | |
| --- | --- | --- | --- | --- | --- |
| (*hkl*) | *d*_exp._ (Å) | *d*_calc._ (Å) | Intensity | Multiplicity | Phase |
| (*211*) | 58.2 | 58.2 | 95.39 | 24 | $\pi$ |
| (*220*) | 50.4 | 50.4 | 26.88 | 12 | $\pi$ |
| (*321*) | 38.1 | 38.1 | 0.29 | 48 | 0 |
| (*400*) | 35.6 | 35.6 | 4.42 | 6 | 0 |
| (*420*) | 31.8 | 31.8 | 0.62 | 24 | 0 |
| (*332*) | 30.4 | 30.4 | 3.84 | 24 | $\pi$ |
| (*422*) | 29.1 | 29.1 | 2.80 | 24 | $\pi$ |
| (*431*) | 27.9 | 27.9 | 0.25 | 48 | $\pi$ |
| (*440*) | 25.2 | 25.2 | 0.09 | 12 | 0 |
| (*611*) | 23.1 | 23.1 | 0.05 | 24 | $\pi$ |
| (*543*) | 20.1 | 20.1 | 0.04 | 48 | 0 |
| Lattice parameter: *a* = 142.4 Å | | | | | |

**Table S3**. Index, experimental and calculated *d*-spacing, intensity (Lorentz and Multiplicity corrected, and normalised to that of the (*211*) peak at 95 ℃ for comparison), and the corresponding calculated lattice parameters of the **4NG** phase formed in **Si3-9** (fresh sample as received) at 135 ℃ on heating from r.t. (**4NG**) to 205 ℃ (*Iso*). The phase combination is used to reconstruct the electron density map ($\rho$-map) of the phase.

| Phase: **4NG**, space group *Ia*$\overline{3}$*d* | | | | | |
| --- | --- | --- | --- | --- | --- |
| (*hkl*) | *d*_exp._ (Å) | *d*_calc._ (Å) | Intensity | Multiplicity | Phase |
| (*211*) | 58.9 | 58.9 | 91.77 | 24 | $\pi$ |
| (*220*) | 51.0 | 51.0 | 27.09 | 12 | $\pi$ |
| (*321*) | 38.6 | 38.6 | 0.30 | 48 | 0 |
| (*400*) | 36.1 | 36.1 | 2.23 | 6 | 0 |
| (*420*) | 32.3 | 32.3 | 0.73 | 24 | 0 |
| (*332*) | 30.8 | 30.8 | 3.00 | 24 | $\pi$ |
| (*422*) | 29.4 | 29.4 | 1.44 | 24 | $\pi$ |
| (*431*) | 28.3 | 28.3 | 0.28 | 48 | $\pi$ |
| (*611*) | 23.4 | 23.4 | 0.03 | 24 | $\pi$ |
| (*541*) | 22.3 | 22.3 | 0.01 | 48 | 0 |
| (*543*) | 20.4 | 20.4 | 0.02 | 48 | 0 |
| Lattice parameter: *a* = 144.3 Å | | | | | |

**Table S4**. Index, experimental and calculated *d*-spacing, intensity (Lorentz and Multiplicity corrected, and normalised to that of the (*211*) peak at 95 ℃ for comparison), and the corresponding calculated lattice parameters of the **4NG** phase formed in **Si3-9** (fresh sample as received) at 155 ℃ on heating from r.t. (**4NG**) to 205 ℃ (*Iso*). The phase combination is used to reconstruct the electron density map ($\rho$-map) of the phase.

| Phase: **4NG**, space group *Ia*$\overline{3}$*d* | | | | | |
| --- | --- | --- | --- | --- | --- |
| (*hkl*) | *d*_exp._ (Å) | *d*_calc._ (Å) | Intensity | Multiplicity | Phase |
| (*211*) | 58.8 | 58.8 | 73.56 | 24 | $\pi$ |
| (*220*) | 50.9 | 50.9 | 21.31 | 12 | $\pi$ |
| (*321*) | 38.5 | 38.5 | 0.21 | 48 | 0 |
| (*400*) | 36.0 | 36.0 | 1.20 | 6 | 0 |
| (*420*) | 32.2 | 32.2 | 0.54 | 24 | 0 |
| (*332*) | 30.7 | 30.7 | 1.94 | 24 | $\pi$ |
| (*422*) | 29.4 | 29.4 | 0.76 | 24 | $\pi$ |
| (*431*) | 28.2 | 28.2 | 0.21 | 48 | $\pi$ |
| (*543*) | 20.4 | 20.4 | 0.01 | 48 | 0 |
| Lattice parameter: *a* = 144.0 Å | | | | | |

1. **Electron density map reconstruction**

**Table S5.** Molecular dimensions of the **Si3-9**.

| Section | Backbone  (Methylsiloxane  +Spacer *n*-C_3_H_6_) | Aromatic core | Aliphatic terminal chains |
| --- | --- | --- | --- |
| Volume ratio*^a^*  (%) | 10 | 44 | 46 |

*^a^* Volume was estimated by using the crystal volume increments method by *Immirzi*.^^[[4]](#endnote-4)^^


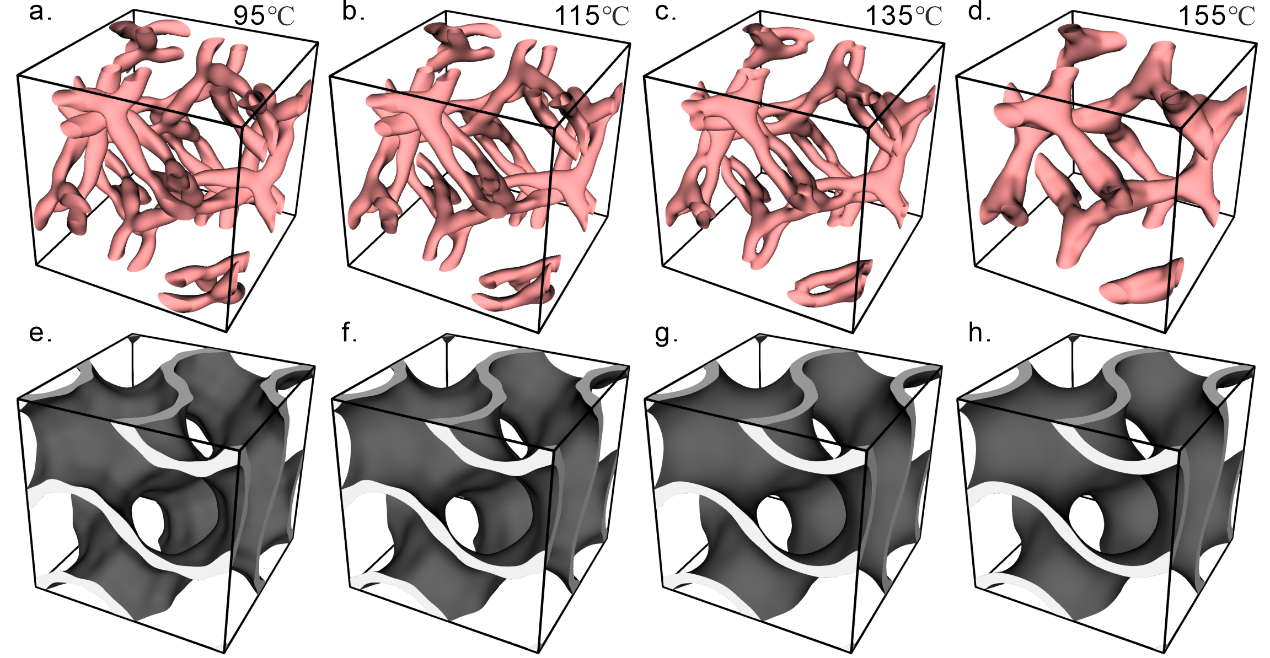


**Figure S7**. Isoelectron surfaces enclose the high 11% and low 18% electron density regions respectively to show the supertwist networks (upside panels) and the aliphatic terminal chains distribution close to the TPMS (downside panels) in the the *Cub*_bi_/*Ia*$\overline{3}$*d* phase in **Si3-9** at various temperatures. **a, e.** at 95 ℃. **b, f.** at 115 ℃. **c, g.** at 135 ℃. **d, h.** at 155 ℃.


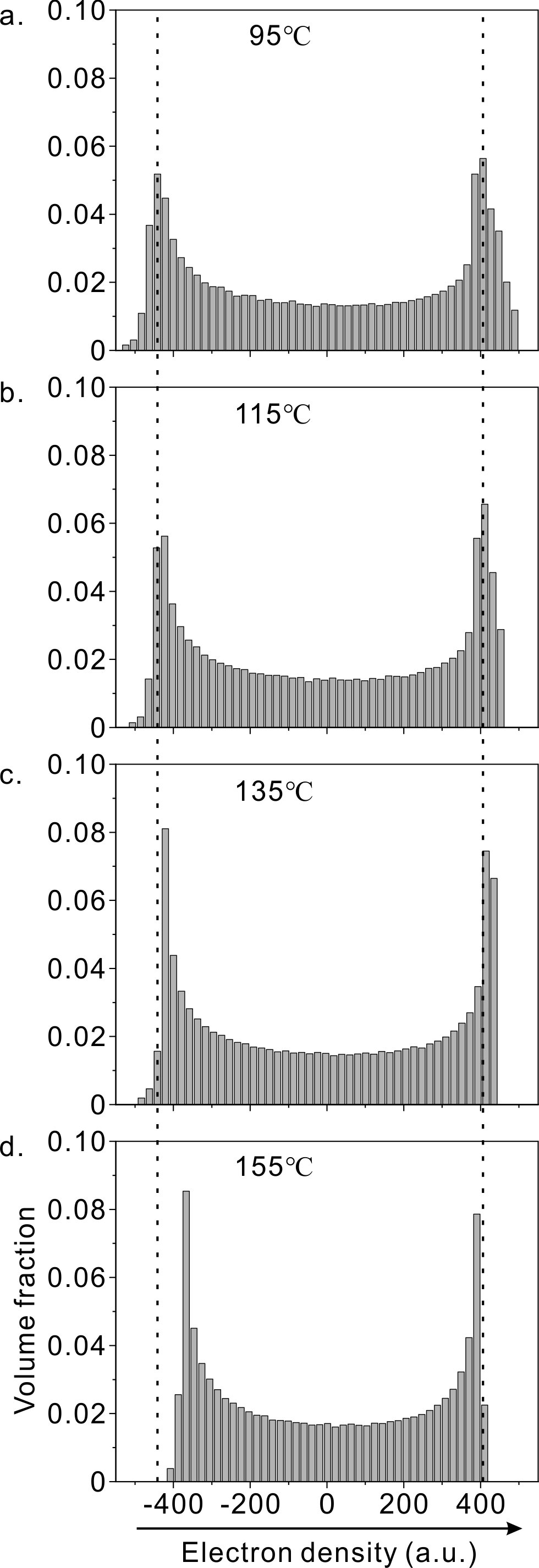


**Figure S8.** Histograms retrieved from the reconstructed electron density map at various temperatures. **a.** at 95 ℃. **b.** at 115 ℃. **c.** at 135 ℃. **d.** at 155 ℃.

**Table S6.** Structural data of the **4NG** phase in **Si3-9**.*^a^*

| *T*  (℃) | *a*_cub_  (Å) | *L_seg_*  (Å) | *m_raft_* | *V*_cell_  (10^6^ Å^3^) | *V*_mono_  (Å^3^) | *n*_cell_ | *n_seg_* | *n*_raft_ |
| --- | --- | --- | --- | --- | --- | --- | --- | --- |
| 95 | 141.6 | 50.1 | 11.1 | 2.839 | 1365 | 1872 | 78.0 | 3.5 |
| 115 | 142.4 | 50.3 | 11.2 | 2.888 | 1365 | 1904 | 79.3 | 3.5 |
| 135 | 144.3 | 51.0 | 11.3 | 3.005 | 1365 | 1981 | 82.6 | 3.7 |
| 155 | 144.0 | 50.9 | 11.3 | 2.986 | 1365 | 1969 | 82.0 | 3.6 |

*^a^* Abbreviations: *T* = temperature; *V*_cell_ = *a*_cub_^3^, is the volume of the unit cell; *L*_seg_ =$\sqrt{2}/4$ *a*_cub_ is the length of the double helical segment between two junction points; m_raft_=*L*_seg_/4.5Å is the number of molecular rafts in each strand between junction points, taking the $\pi-\pi$ stacking distance between rafts of monomers to be 4.5 Å;^[[5]](#endnote-5)^ *V*_mono_ is the monomer volume calculated according to the crystal volume increments method by Immirzi;^4^ *n*_cell_ = 0.9 *V*_cell_ / *V*_mono_, is the monomer number in a unit cell, the constant 0.9 is a correction factor for the packing density difference between LC and crystalline states; *n*_seg_ = *n*_cell_/24, is the monomer number in each double helical segment between junction points; *n*_raft_=*n*_seg_/(2**m*_raft_) is the number of monomers in each raft of a helical strand.

1. **Selection of phase combinations in the reconstruction of electron density maps**

As indicated in section S1.6, in reconstructing the electron density maps the phase of structure factor $\phi_{hkl}$ cannot be determined from X-ray diffraction experiment directly and must be determined instead on the merit of the electron density maps reconstructed. The fact that the $Ia\bar{3}d$ space group is centrosymmetric is helpful, as the structure factors are real numbers so that $\phi_{hkl}$ is either 0 or $\pi$. We use various criteria in selecting the phase combination for the reconstruction of the electron density maps. The main ones are the volume vs electron density histograms (see Figs. S8 and S9) and ensuring smoothness and continuity of the main regions of the unit cell, effectively adopting the crystallographic principle of maximum entropy.

Take the maps at 95°C on heating as an example, there 13 reflections were used but it should be noted that some of them are very weak hence have only a very minor effect on the map. Apart from the two strongest peaks, (211) and (220), there are only three other reflections, (400), (332) and (422), that have relative intensities higher than 1% of that of the (211) peak. Therefore the phase choices of the other 8 peaks will not make any substantial changes to the maps or change our interpretation of them in terms of the molecular structural model, even though their inclusion would help improve the resolution and quality of fit to the structural model. At the same time, there is no doubt in the phases of the two strongest peaks (211) and (220) as they were both set to be $\pi$ by the underlying gyroid structure. Consequently our job is essentially reduced to decide on the signs of the three other reflections, with only 8 possible phase combinations. All 8 maps are shown in Figure S9a-h respectively below by isoelectron surfaces enclosing the high (25% of the aromatic volume, in red) and low (33% of the aliphatic end-tails, in blue) electron density regions, and the corresponding volume vs electron density histograms. Six of the maps can be easily dismissed as the low electron density regions do not cover the minimum surface evenly and smoothly as expected in a gyroid phase. The two remaining maps can be decided on the basis of the continuity of the high electron density regions and the histogram. Consequently phase combination (−−−−−) can be dismissed as the high electron density regions are disconnected, and its histogram show extra tails at both the high the low electron density ends compared to phase combination (−−+−−).


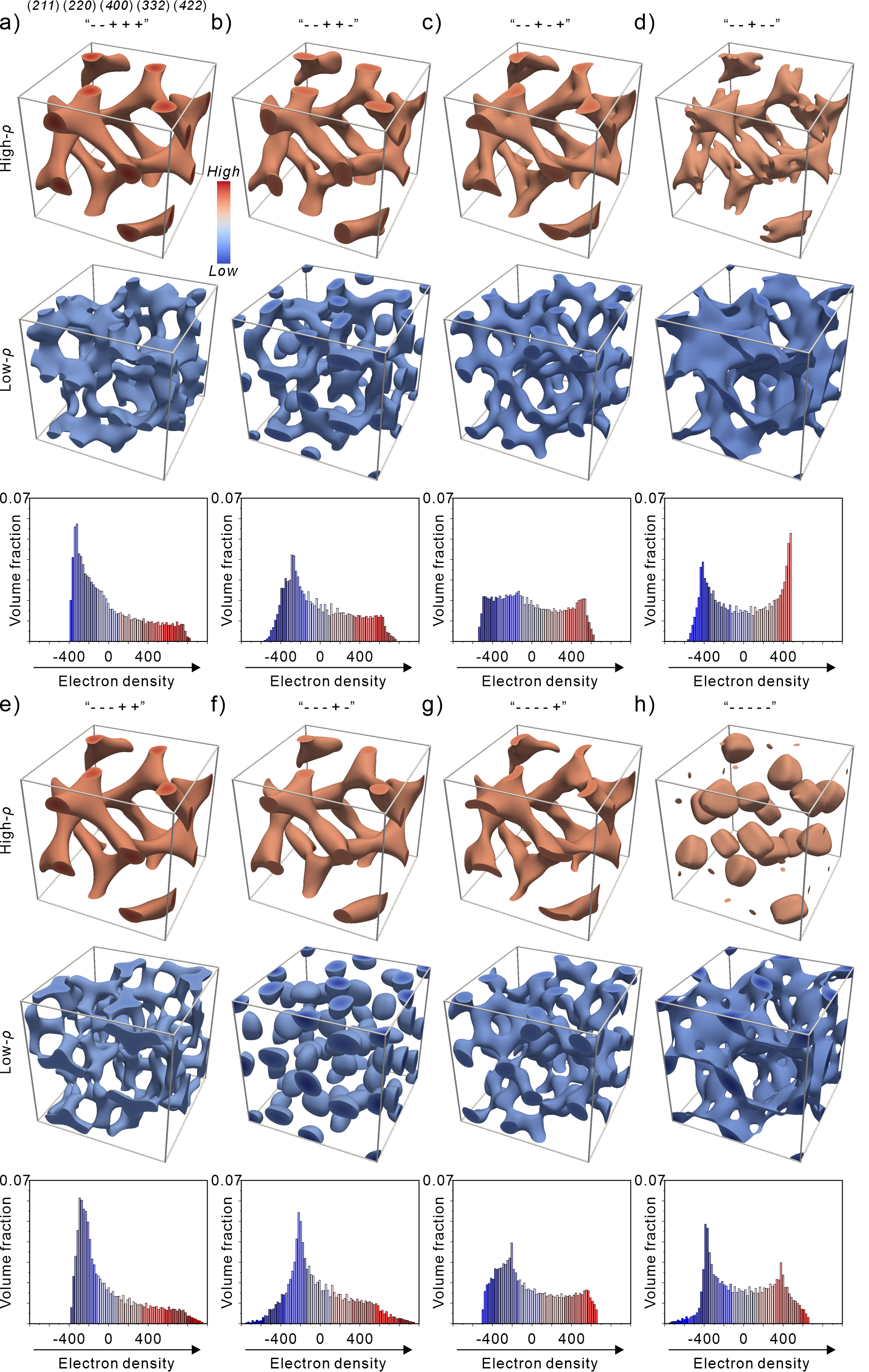


**Figure S9.** (a-h) Reconstructed electron density maps from 5 strongest diffraction peaks, (211), (220), (400), (332) and (422), of **Si3-9** at 95°C on heating, using 8 different phase combinations. For each phase combination the high electron density regions (enclosing 25% of the aromatic volume, in red), and the low electron density regions (33% of the volume of the aliphatic end-tails, in blue) are shown along with its histogram. The phase combination is shown on top of each panel. Phase combination “− − + − −” is selected as it shows connected high electron density regions, smooth low electron density regions covering the minimum surface, and the narrowest histogram showing peak volumes at both the high and low ends of the electron density, as expected from a gyroid structure.

**References**

1. X. B. Zeng, G. Ungar, M. Impéror-Clerc, *Nature Mater.* **2005**, *4*, 562–567. [↑](#endnote-ref-1)
2. V. Percec, J. Heck, G. Ungar, *Macromolecules* **1991**, *24*, 4957-4962. [↑](#endnote-ref-2)
3. X. B. Zeng, L. Cseh, G. H. Mehl, G. Ungar, *J. Mater. Chem.* **2008**, *18*, 2953-2961. [↑](#endnote-ref-3)
4. A. Immirzi, B. Perini, *Acta Cryst.* **1977**, *A33*, 216-218. [↑](#endnote-ref-4)
5. C. Dressel, T. Reppe, S. Poppe, M. Prehm, H. Lu, X. B. Zeng, G. Ungar, C. Tschierske, *Adv. Funct. Mater.* **2020**, *30*, 2004353. [↑](#endnote-ref-5)
